# Novel stem cell therapy for cerebral palsy using stem cells from human exfoliated deciduous teeth

**DOI:** 10.1186/s13287-025-04828-y

**Published:** 2026-01-23

**Authors:** Takahiro Kanzawa, Atsuto Onoda, Azusa Okamoto, Xu Yue, Ryoko Shimode, Yukina Takamoto, Sakiko Suzuki, Kazuto Ueda, Ryosuke Miura, Toshihiko Suzuki, Naoki Tajiri, Shinobu Shimizu, Saho Morita, Hiroshi Yukawa, Hiroshi Kohara, Noritaka Fukuda, Yasuyuki Mitani, Hideki Hida, Yoshiyuki Takahashi, Yoshiaki Sato

**Affiliations:** 1 Division of Neonatology, Center for Maternal-Neonatal Care, Nagoya University Hospital, 65 Tsurumai-cho, Showa-ku, Nagoya, Aichi 466-8560 Japan; 2 Department of Pediatrics, Nagoya University Graduate School of Medicine, 65 Tsurumai-cho, Showa-ku, Nagoya, Aichi 466-8560 Japan; 3 Department of Toxicology and Health Science, Faculty of Pharmaceutical Sciences, Sanyo-Onoda City University, Daigakudori 1-1-1, Sanyo-Onoda, Yamaguchi 756-0884 Japan; 4 Department of Neurophysiology and Brain Science, Nagoya City University Graduate School of Medical Science, 1 Kawasumi, Mizuho-cho, Mizuho-ku, Nagoya, 467-8601 Japan; 5 Department of Advanced Medicine, Nagoya University Hospital, 65 Tsurumai-cho, Showa-ku, Nagoya, Aichi 466-8560 Japan; 6 Department of Biomolecular Engineering, Graduate School of Engineering, Nagoya University, Furo-cho, Chikusa-ku, Nagoya, 464-8601 Japan; 7 Research Institute for Quantum and Chemical Innovation, Institutes of Innovation for Future Society, Nagoya University, Furo-cho, Chikusa-ku, Nagoya, 464-8601 Japan; 8 Quantum Regenerative and Biomedical Engineering Team, Institute for Quantum Life Science, National Institutes for Quantum Science and Technology, 4-9-1 Anagawa, Inage-ku, Chiba, 263-8555 Japan; 9 S-Quatre Corporation, 3-8-3 Nihombashihoncho, Chuo-ku, Tokyo, 103-0023 Japan

**Keywords:** Stem cells from human exfoliated deciduous teeth, Cerebral palsy, Hypoxic-ischemic encephalopathy, Neurogenesis, Hepatocyte growth factor, PI3K-Akt signaling pathway, Neonatal brain injury, Behavioral recovery, Quantum dots, Neural stem cells

## Abstract

**Background:**

Effective treatments for cerebral palsy caused by Hypoxic-ischemic encephalopathy are urgently needed. Current therapies primarily include prevention or acute intervention, leaving a major gap in the options for reversing established neurologic damage. Because of their ease of collection and unique trophic factor profile, stem cells from human exfoliated deciduous teeth (SHED) are promising candidates for cell-based therapy targeting neurological disorders. In this study, we examined the therapeutic potential of SHED in a rat model of cerebral palsy, focusing on neurogenic and functional recovery.

**Methods:**

Hypoxic–ischemic encephalopathy was induced in neonatal rats using the Rice–Vannucci method. Rats with motor impairments received intravenous SHED injections, whereas the control group received a vehicle solution. Behavioral tests assessed motor coordination and cognitive performance. Proteomic analyses and immunohistochemistry were performed to examine the underlying mechanisms. The migration and biodistribution of SHED were tracked using quantum dot-labeled SHED with in vivo imaging. Neural stem cells were cocultured with SHED to evaluate neurogenesis, followed by RNA sequencing and the analysis of trophic factors in the conditioned media.

**Results:**

SHED treatment significantly ameliorated motor coordination, memory, and learning. Proteomic analysis revealed increased expression of proteins associated with neurogenesis in the SHED group. Histopathologic evaluations revealed enhanced neurogenesis in the hippocampal dentate gyrus and subventricular zone 2 weeks posttreatment, with increased NeuN-positive cells in the hippocampus and cortex at ten weeks. In vivo imaging revealed the migration of quantum dot-labeled SHED to the brain. Neural stem cells co-cultured with SHED in vitro exhibited higher proliferation rates. The SHED-conditioned medium contained increased levels of hepatocyte growth factor (HGF), and HGF-neutralizing antibodies suppressed the enhanced cell proliferation. RNA sequencing revealed significant alterations in genes associated with the PI3K–Akt signaling pathway.

**Conclusions:**

SHED treatment ameliorated motor, memory, and learning impairment in a rat model of cerebral palsy. These improvements were accompanied by enhanced neurogenesis, likely mediated by HGF secretion and activation of the PI3K–Akt signaling pathway. SHED is a promising candidate for postsymptom-onset treatment of cerebral palsy. Further studies to confirm these findings and examine the clinical utility of SHED are warranted.

**Supplementary Information:**

The online version contains supplementary material available at 10.1186/s13287-025-04828-y.

## Background

Over the past 20–30 years, perinatal care has significantly improved, leading to a decrease in the neonatal death rate. However, the incidence of cerebral palsy has remained steady [1], occurring in 2–3 cases per 1000 live births [2]. The main cause of cerebral palsy is perinatal Hypoxic-ischemic encephalopathy (HIE). Despite this, the only available standard treatment for moderate or severe HIE is therapeutic hypothermia, and this treatment is insufficient [3]. Recent reports have demonstrated the efficacy of stem cell therapy for the acute/subacute phase of HIE [4–9]. However, it is important to address developmental delays in children during the chronic phase, as some children with seemingly low disability levels at birth may later develop significant developmental issues [10].

Clinical trials performed using cord blood, bone marrow-derived mesenchymal stromal cells (BMMSC), and neural stem cells (NSC) have been reported for stem cell therapy in the chronic stages of cerebral palsy [11–15]. Although clinical trials have been conducted to determine the efficacy of stem cell treatments in chronic-phase patients, there is still a lack of mechanistic evidence supporting their therapeutic effects.

Stem cells from human exfoliated deciduous teeth (SHED) are harvested from the dental pulp of human deciduous teeth [16]. SHED can mediate its effects through paracrine mechanisms, in which soluble factors, such as cytokines, chemokines, extracellular vesicles, and growth factors, are released into the local tissue microenvironment [17]. These factors regulate various biological processes, such as neuroprotection [18–20], anti-inflammation [21–23], angiogenesis [24–27], and promote cell proliferation and differentiation, thereby contributing to tissue regeneration [17, 21, 28–34]. Previous studies have shown the therapeutic potential of SHED in models of neurological diseases. For example, they can secrete signal transduction proteins, neurotrophic factors, and extracellular vesicles that repair damaged neurons in Parkinson’s disease models [35, 36]. In Alzheimer’s disease models, SHED can ameliorate symptoms by enhancing glucose metabolism and restoring mitochondrial function [37, 38]. Early SHED treatment also inhibits apoptosis, oxidative stress, and microglial activation in HIE models, which improves histological and behavioral outcomes [39, 40]. Furthermore, SHED enhances cognitive function in animal models of chronic cerebral ischemia by inhibiting neuronal apoptosis [41]. Single-cell transcriptome analysis revealed that SHED exert more robust immunomodulatory properties compared with human BMMSCs or human umbilical cord-derived mesenchymal stromal cells [42]. In addition, the cytokines secreted by SHED and BMMSC showed different features with SHED secreting higher levels of hepatocyte growth factor (HGF), matrix metalloproteinase-3, bone morphogenetic protein-7, and stromal cell-derived factor 1 [43]. Of these, HGF activates the phosphoinositide 3-kinase (PI3K)–Akt signaling pathway through its receptor, Met, which results in the phosphorylation and activation of Akt. As a key downstream effector of the PI3K pathway, Akt plays a central role in regulating cell proliferation, survival, and metabolism. Full activation of Akt requires phosphorylation at Thr308 by 3-phosphoinositide-dependent protein kinase-1 (PDK1), as well as Ser473 by the mammalian target of rapamycin complex 2 (mTORC2) [44]. HGF stimulates mTORC2 activity, thereby promoting the Ser473 phosphorylation of Akt, resulting in its maximal activation [45]. This signaling cascade inhibits the nuclear translocation of the cell cycle inhibitor p27 Kip1, thereby promoting NSC proliferation and contributing to neurogenesis [46, 47]. The ethical issues associated with harvesting teeth are minimal, as SHED uses deciduous teeth that are normally discarded [33], and autologous transplantation is possible. Furthermore, similar to other MSCs, allogeneic cells can also be administered intravenously due to their low immunogenicity and immunomodulatory capacity [48]. SHED’s availability and biological characteristics make it a promising candidate as a stem cell source for neurological diseases, especially, the chronic phase of neonatal brain injury (cerebral palsy).

In this study, we investigated whether the administration of SHED in the chronic phase of a rat model of HIE with neurological symptoms ameliorated these symptoms. Furthermore, through in vitro experiments, we compared its effects on NSC with those of other stem cells and elucidated the mechanisms underlying its efficacy.

## Methods

### Animals

The study was designed as a randomized controlled trial to evaluate the effects of SHED in the chronic phase of a rat model of HIE presenting with neurological symptoms. The primary outcome measure was the amelioration of motor deficits in cerebral palsy models, assessed by the improvement in scores from the horizontal ladder test following SHED administration. All experiments were approved by the Animal Care and Use Committee of Nagoya University School of Medicine (Nagoya, Japan; permit No.M220140-002, M220142-003) and were conducted in accordance with the Regulations on Animal Experiments in Nagoya University. Additionally, the work has been reported in line with the ARRIVE guidelines 2.0 (Animal Research: Reporting in Vivo Experiments). The experimental unit was the individual rat. In order to minimize individual variation due to the sexual cycle, male Wistar/ST rat pups were used in this study (*n* = 90). Experimental rats were obtained from Japan SLC Inc. (Shizuoka, Japan). Neonatal rats were housed with their dams in a temperature-controlled room (23 °C) with a 12-h light/dark cycle and remained together until weaning on postnatal day 21. After weaning, the animals were housed under the same conditions with access to food and water ad libitum. The pups were selected based on general health status and body weight within − 2 standard deviations (SDs) of the litter mean on postnatal day 7. Any animals showing signs of malformation, severe underdevelopment, or illness were excluded from the study.

### SHED cell culture

Stem cells from human exfoliated deciduous teeth (SHED) were obtained from Kidswell Bio Corporation (Tokyo, Japan). In all experiments, these cells were used in passages 4–7. The characteristics of SHED (including its surface proteins and multilineage differentiation potential) are available at https://www.summitpharma.co.jp/japanese/service/s_ATCC_msc.html. The cells were cultured MesenCult™-hPL Medium Kit (STEMCELL Technologies, Vancouver, Canada) containing 1% GlutaMAX™ supplement (Thermo Fisher Scientific, Waltham, MA, USA). Cells were maintained at 37 °C with 5% CO_2_ by replacing the medium with fresh medium at 2-day intervals.

### Hypoxic-ischemic insult and cerebral palsy model

For the perinatal rat HIE model, 7-days-old Wistar/ST rats were used as described before [49, 50]. In brief, the rat pups were anesthetized with isoflurane, and the left common carotid artery was double-ligated and sectioned, after which the rats were placed in an incubator at 37 °C and 8% hypoxia for 60 min. If severe neurological dysfunction, persistent seizures, or inability to sustain growth independently was observed during the developmental period following hypoxic exposure, the experiment was terminated, and the animals were humanely euthanized. Euthanasia of animals was performed using carbon dioxide (CO₂). To evaluate their motor function, the horizontal ladder test was then performed at the age of 4 weeks as previously reported [51, 52]. The horizontal ladder apparatus consisted of a 1-m-long by 4-cm-wide ladder equipped with two transparent Plexiglas walls, each perforated with apertures at 1-cm intervals. It was positioned 75 cm above the floor, with an empty cage at the starting point and a home cage at the endpoint. To evaluate hindlimb step function, the rats were trained for 3 consecutive days to traverse the 1-m ladder with rungs spaced evenly at 1-cm intervals, moving at a constant speed toward the home cage. On the test day, to assess coordinated hindlimb movement, the spacing between the rungs was varied randomly between 1 and 3 cm. The rats were video-recorded as they traversed the ladder three times. The hind-limb gait of each rat was graded on a 0–4 scale (Additional File 2: Supplemental Table 1). For every attempt, the step scores were summed and divided by the number of hind-limb steps to obtain a score per step. Each animal completed three independent crossings, and the mean of these three scores was designated as its representative gait score. For gait scores lower than 1 SD below the mean of the sham-operated group, the rats were candidates for the cerebral palsy model and were used in the subsequent experiments.

### Assignment of groups and SHED administration

Cerebral palsy model rats were randomly and evenly allocated to two groups according to the results of the horizontal ladder test to ensure balanced baseline motor function between the groups. A computer-generated randomization sequence was employed to ensure unbiased group allocation. SHED (1 × 10⁶ cells/body, equivalent to 1 × 10⁵ cells/g body weight, in 0.5 mL Ringer’s lactate solution) was administered via the tail vein at 5, 7, and 9 weeks of age without immunosuppressive agents in the SHED group, whereas the vehicle and sham groups received vehicle alone (0.5 mL of Ringer’s lactate solution). The cell dose was determined based on previous studies using similar cell numbers for comparable disease models [39]. The administration schedule was designed based on the expected timing for clinical application, in which 5 weeks of age in the animal model corresponds to the preadolescent stage in humans [53]. A 2-week interval was adopted to consider in vivo safety and for migration and biodistribution analysis. Therefore, this schedule ensured sufficient clearance of the previous dose before each subsequent administration, minimizing the risk of unintended accumulation. To minimize potential confounders, treatments and measurements were conducted in a randomized order.

### Shuttle avoidance test

Shuttle avoidance tests [54] were conducted to assess memory and learning in rats. Each rat underwent 6 sets of 10 sessions, with each session including a 5-second-long alert and an electric shock. Rats had to escape to the other side of the shuttle box to avoid the shock. The avoidance rate was measured using the number of successful escapes. The avoidance rates of the first half (sets 1–3) and second half (sets 4–6) were compared with each other.

### Cylinder test

Cylinder tests were performed to assess forelimb use preference, as previously reported [50]. Forelimb use preference was calculated as follows: ((nonimpaired - impaired)/(nonimpaired + impaired + both)). The mean value for each rat was used for statistical analyses. The investigators performing the outcome assessments were blinded to group allocation.

### Distribution of SHED in the brain

To investigate the engraftment of SHED after intravenous administration, we used quantum dots (QDs) (Qdot 800 ITKCarboxyl Quantum Dots, Thermo Fisher Scientific) as previously described [39, 55, 56]. Then, 8 nM of QDs was added to cultured SHED, and QDs-labeled SHED was administered via the tail vein of the cerebral palsy model rat (1 × 10^6^ cells/body). Thereafter, brains were harvested at four time points (1 h, 24 h, 48 h, and 1 week after the administration of QDs-labeled SHED). Brain samples were observed with an IVIS^®^ imaging system (Revvity, Waltham, MA) and the fluorescence intensity was measured. The fluorescence intensity ratio (SHED/Vehicle) at each time point was calculated. To visualize the three-dimensional deposition of QD-labeled SHED in the brain, we cleared the explanted brains as follows [57]:

Brain tissues fixed in 4% PFA-PBS were washed with PBS, immersed in CUBIC-L (a reagent for delipidation), and incubated for 1 week. The tissues were incubated in PBS with 1% Anti-Actin, α-Smooth Muscle-FITC antibody (Sigma-Aldrich, Inc., St. Louis, MO), 1% RedDot 2 (Biotium, Inc., Fremont, CA), and 0.25% Blocker Casein (Thermo Fisher Scientific) for 5 days. The tissues were then immersed in CUBIC-R, a refractive index (RI)-matching reagent diluted 1:1 in water, for a day. RI matching was then completed by immersing the organs in CUBIC-R for 2 days. The cleared brains were observed with a light-sheet microscope (UltraMicroscope II, Miltenyi Biotech, Bergisch Gladbach, Germany), and the captured images were 3D-rendered and visualized using Imaris (Oxford Instruments plc, Abingdon, UK).

### Proteomic analysis

Proteins were extracted from the brain after SHED administration, and proteomic analyses were performed as previously reported [58]. Brains were collected 2 weeks after SHED administration to cerebral palsy models. The samples were frozen using liquid nitrogen, homogenized using Multi-bead shocker (Yasui Kikai Co., Ltd., Osaka, Japan), and total proteins were extracted with the T-PER protein extraction reagent (Takara Bio. Inc., Shiga, Japan) containing a protease inhibitor cocktail (Sigma-Aldrich). The protein lysate supernatants were labeled with the Tandem Mass Tag™ system (Sixplex TMT, Thermo Fisher Scientific) for liquid chromatography/tandem mass spectrometry (LC/MS/MS). LC/MS/MS was conducted using an Orbitrap Fusion mass spectrometry system (Thermo Fisher Scientific) coupled with an UltiMate 3000 RSLCnano LC system (Dionex, Amsterdam, The Netherlands) and a nano-electrospray ionization source. A nano-capillary column (150 mm × 75 μm, Nikkyo Technos, Tokyo, Japan) was used for separation. Peptides were separated by reversed-phase chromatography using a linear gradient from 5% to 40% of solvent B (0.1% formic acid in 95% acetonitrile) in solvent A (0.1% formic acid in 2% acetonitrile) for 100 min at a flow rate of 300 nL/min.

Expression levels of the detected proteins were statistically compared between the SHED and vehicle groups. The false discovery rate (FDR) was calculated using the Storey method based on the p-value. The proteins affected by SHED administration were extracted using threshold levels set at *p* < 0.05 and FDR < 0.10. The extracted protein profile was used for the functional annotation analysis on the Database for Annotation, Visualization, and Integrated Discovery 6.8 (DAVID 6.8). To identify functionally significant gene clusters, a threshold of Enrichment Score (ES) >2.0 was applied. This cutoff is commonly used in gene ontology and pathway enrichment analyses, and reflects an empirically supported criterion for biological relevance. An ES >1.3 corresponds to *p* < 0.05, and while often accepted as statistically significant, a higher threshold (ES >2.0) was used in several studies to identify clusters with stronger enrichment and greater biological significance [59, 60].

For proteomic analysis, samples matched for severity were selected based on the results of the horizontal ladder test to minimize variability within each group. Previous studies have demonstrated that proteomic signatures are consistent among samples with comparable disease severity [61], which supports the robustness of our sample selection strategy.

### Tissue preparation and immunohistochemistry

Brain tissue samples were collected at two points, which were the early (2 weeks) and late (10 weeks) time points after SHED administration. For the evaluation at the early time point, 50 mg/kg of bromodeoxyuridine (BrdU Roche, Basel, Switzerland) was administered intraperitoneally for three consecutive days following the last administration of SHED. Immunohistological staining was performed as previously reported [39]. All the rats were administered three types of mixed anesthetic agents (medetomidine, midazolam, and butorphanol) intraperitoneally to anesthetize them deeply. Perfusion fixation was then performed using 4% paraformaldehyde, and serial coronal sections were made. After antigen retrieval, the sections were incubated overnight at 4 °C with primary antibody [mouse anti-BrdU; 1:200, abcam, Cambridge, UK, rabbit anti-Doublecortin (DCX); 1:200, abcam, mouse anti-NeuN; 1:200, Merck, Boston, MA, mouse anti- SRY-Box Transcription Factor 2 (SOX2); 1:100, Santa Cruz, Texas, USA, or rabbit anti-Phospho-Akt (Ser473); 1:200 CST, MA, USA] in PBS containing 4% donkey serum (Jackson Immuno Research Laboratories, Baltimore Pike, PA, USA) and 0.1% Triton X-100. The sections were subsequently incubated for 1 h at room temperature with Alexa Fluor-conjugated donkey anti-rabbit (488 or 555) or anti-mouse (488 or 555) secondary antibody (1:500; Thermo Fisher Scientific), and mounted using ProLong Gold Antifade Reagent (Thermo Fisher Scientific). Alternatively, sections were incubated with the appropriate biotinylated secondary antibody for 60 min, followed by 3% H_2_O_2_ for 10 min. Binding was visualized using a Vectastain ABC kit (Vector Laboratories, Burlingame, CA, USA) with peroxidase detection for 12 min (0.12 mg/mL 3,3′- diaminobenzidine, 0.01% H_2_O_2_ and 0.04% NiCl_2_).

### Cell counting

Sections were prepared at 5 μm and stained at 50-section intervals to count the cells that tested positive in each immunostaining. The NeuN-positive cells were counted throughout the hippocampal dentate gyrus and cortex using Stereo Investigator^®^ version 10 stereology software (MicroBrightField Europe EK, Magdeburg, Germany [39]). Quantitation was done using the optical fractionator probe of Stereo Investigator. A 25 μm × 25 μm counting frame was applied to an 80 μm × 190 μm sampling grid in the hippocampal dentate gyrus. For the cortex, a 50 μm × 50 μm counting frame was applied to an 800 μm × 800 μm sampling grid. The optical dissector height was set to 5 μm, with no guard zones at the top or bottom of each section. Positive cells were counted using a 40× objective lens, and Gundersen coefficients of error (m = 1) were ≤ 0.10 for all estimates.

To determine the number of immature neurons that proliferated and differentiated after SHED administration in the hippocampal dentate gyrus and striatum, the DCX positivity rate was determined by counting the BrdU-positive cells and BrdU/DCX double-positive cells in each region in two representative sections. To select these sections, systematic random sampling was performed to ensure spatial uniformity. For each animal, two reference sections were selected at fixed rostrocaudal intervals of 500 μm. The first section was randomly selected within the initial 250 μm block, whereas the second section was taken exactly one interval caudally. The total number of BrdU/DCX double-positive cells was estimated by multiplying the DCX positivity rate by the total number of BrdU-positive cells in each region, as previously described [62].

### Measurement of proliferation and phosphorylation in neural stem cells in vitro

Adult Rat Hippocampal Neural stem cells (NSC, SCR022, Merck) were plated on plates coated with Poly-L-ornithine (P3655, Merck) and Laminin (CC095, Merck), and cultured in DMEM/F12 (Thermo Fisher Scientific) containing the 2% B-27 supplement (Thermo Fisher Scientific), 1% GlutaMAX™ supplement (Thermo Fisher Scientific), and 20 ng/mL fibroblast growth factor basic protein (GF003, Merck). The initial seeding density of the NSCs was 1 × 10^4^ cells per well. SHED or bone marrow-derived human mesenchymal stromal cells (BMMSCs, PT-2501, Lonza, Basel, Switzerland) or human Dermal Fibroblasts (DFs, ZEN-Bio, North Carolina, USA) were seeded into Transwell inserts (Falcon™ Cell Culture Inserts for 24-well plates with a 0.4 μm PET transparent membrane, Corning, NY, USA), and cultured using their respective media as described above for SHED, BMMSC, or the Dermal Fibroblast Culture Medium (DF-1, ZEN-Bio) for DF. Twenty-four hours after seeding the cells, the medium was changed to MEM α, nucleosides, no phenol red (Thermo Fisher Scientific), and then the inserts were transferred to wells in which NSCs were cultured. In the control group, only the inserts without seeded cells were transferred to the wells. Coculture was maintained for 48 h, and the duration was determined based on our migration and biodistribution analyses, which demonstrated that intravenously administered cells remain in the brain for at least 48 h. After 48 h of co-culture, NSCs were fixed for 30 min at room temperature with 3% paraformaldehyde containing 1% sucrose, 1 mmol/L MgCl_2_, and 0.1 mmol/L CaCl_2_. The fixed cells were incubated overnight at 4°C with primary antibody (mouse anti-SOX2; 1:200, Santa Cruz, rabbit anti-S100; 1:1000, abcam, rabbit anti-Ki67; 1:200 abcam, rabbit anti-Phospho-Akt (Ser473); 1:600 CST, rabbit anti-Phospho-Akt (Thr308); 1:1600 CST) in PBS containing 4% donkey serum and 0.1% Triton X-100. The cells were incubated with Alexa Fluor-conjugated secondary antibodies: donkey anti-rabbit IgG 488 and donkey anti-mouse IgG 555 or anti-rabbit IgG 555 (all 1:500) for 1 h at room temperature and stained with Hoechst^®^ 33342 (1:300, DOJINDO LABORATORIES, Kumamoto, Japan) for 30 min at room temperature. We counted the positive cells in all wells using the same methods described above. A 30 μm × 30 μm counting frame was applied to an 850 μm × 850 μm sampling grid. As previously described [63], the NSC growth rate was defined as follows: the number of SOX2-positive and S100-negative cells after coculture divided by the number of seeded cells. Proliferation was also evaluated by calculating the proportion of Ki67-positive cells within the total cell population. Akt phosphorylation was quantified by measuring the intracellular fluorescence intensity of Phospho-Akt (Ser473) and Phospho-Akt (Thr308) using the Intensity Profile function of cellSense software (version 4.2, Olympus, Tokyo, Japan). For each experimental group, 10 cells were randomly selected from three independent wells.

### RNA sequence

Total RNA was extracted from the NSC cultures 48 h after being cocultured with SHED, BMMSC, or DF using the RNeasy Mini Kit (Qiagen Inc. Venlo, Netherlands) per the manufacturer’s protocol.

Messenger RNA was purified from total RNA using poly-T oligo-attached magnetic beads, fragmented, and converted to cDNA. For non-directional libraries, the steps involved included end repair, A-tailing, adapter ligation, size selection, amplification, and purification. Directional libraries included a supplementary USER enzyme digestion step. Libraries were checked with Qubit, real-time PCR, and bioanalyzer. Reference genomes were indexed with Hisat2, and reads were aligned using Hisat2. Reads were counted with feature Counts, and FPKM values were calculated.

Differential expression [64] analyses were performed with DESeq2Rpackage (1.20.0), adjusting p-values using Benjamini and Hochberg’s method, with genes having adjusted p-values of ≤ 0.05 being considered differentially expressed. GO enrichment analyses [65] of differentially expressed genes were conducted using the clusterProfiler R package, correcting for gene length bias. GO terms with corrected p-values of < 0.05 were considered significantly enriched. The clusterProfiler R package was also used to test the statistical enrichment of differentially expressed genes in KEGG [66] pathways.

NSCs used for RNA-seq were clonally derived and cultured under uniform in vitro conditions. These cells shared identical genetic backgrounds and were maintained in strictly standardized environments to minimize biological variability. NSC cultures have been shown to exhibit low inter-sample heterogeneity under comparable conditions [67, 68].

### Evaluation of trophic factors

Forty-eight hours after co-culturing with SHED, BMMSC, or DF, NSC-conditioned medium samples were collected, and 120 cytokines were measured using RayBio Human Cytokine Antibody Array C 1000 (RayBiotech, Peachtree Corners, GA) according to the manufacturer’s protocol. The primary purpose of using this human cytokine array was to detect cytokines secreted by human-derived cells in coculture with rat NSCs. Chemiluminescence imaging was performed using the ChemiDoc Touch MP imaging system (Bio-Rad, Hercules, CA). Data were presented as the ratio of the chemiluminescence intensity measured using the each conditioned medium to that measured using conditioned medium from NSCs cultured alone (Control). To minimize the effect of cross-species reactivity, the chemiluminescence intensities obtained from the co-culture conditioned medium were normalized to those from the conditioned medium of the control. The HGF concentration in the appropriate conditioned medium was confirmed using an Enzyme-Linked Immunosorbent Assay (ELISA) kit (Human HGF Quantikine ELISA Kit, RayBiotech).

### Neutralization of enhanced neural stem cell proliferation

AncBE4max [69] cDNA and the corresponding sgRNAs were synthesized by Genscript (Piscataway, NJ, USA). The mRNA was transcribed using an in vitro transcription kit (Takara Bio, Shiga, Japan). Lipid nanoparticles (LNPs) encapsulating the mRNA were prepared as previously described [70], and HGF-knockout SHED (HGF-KO-SHED) were established (Additional File 3: Supplementary Fig. 1).

NSC co-cultures were established as described above and divided into the following four groups: SHED, HGF-KO-SHED, HGF-neutralizing antibody, and control. In the neutralizing antibody group, goat anti-human HGF neutralizing antibody (10 µg/mL; R&D Systems, Minneapolis, MN, USA) was added to the medium of NSCs and SHED at the beginning of coculture. A concentration of 10 µg/mL was selected to ensure effective neutralization of HGF, based on a previous study that used 5 µg/mL in an NSC proliferation assay [71]. NSC proliferation and Akt phosphorylation under the four different conditions were measured 48 h after coculture was initiated using the methods described above.

A rescue experiment was conducted, in which recombinant human HGF protein (25 ng/mL; Proteintech, Rosemont, IL, USA) was added to the culture medium of NSCs and HGF-KO-SHED at the beginning of coculture. Cell proliferation and Akt phosphorylation were evaluated after 48 h using the same procedures.

### Microscopy

Immunohistochemical and immunocytochemistry images were acquired using an inverted fluorescence microscope (IX83, Olympus Corporation, Tokyo, Japan) equipped with a DP73 camera (Olympus Corporation). For tissue staining, DCX/BrdU was imaged using a 10× objective lens. NeuN was imaged at low (10× objective lens) and high (40× objective lens) magnification. SOX2/p-Akt (Ser473) was imaged using a 10× objective lens. For cultured cells, SOX2/S100 and Ki67 were imaged using a 10× objective lens, and p-Akt was imaged using a 40× objective lens. These images were used for the quantitation of cell numbers and fluorescence intensity.

Representative images of DCX/BrdU and SOX2/p-Akt (Ser473) in tissue sections, as well as p-Akt in cultured cells, were imaged using a confocal laser scanning microscope (Ti-E A1R, Nikon Corporation, Tokyo, Japan). Confocal images of DCX/BrdU and SOX2/p-Akt (Ser473) at low magnification were captured using a 10× objective lens. High-magnification images were captured using a 100× water-immersion objective lens. Confocal images of p-Akt in cultured cells were imaged using a 60× water-immersion objective lens. No digital zoom was applied in any case. Image analysis was conducted using cellSens software Ver. 4.2 (Olympus Corporation) or Fiji software (ImageJ distribution, NIH, Bethesda, MD, USA) [72].

### Statistical analyses

Statistical analyses were performed by using GraphPad Prism software version 10 (GraphPad Software, San Diego, CA). Prior to statistical analysis, the data were tested for normality and homogeneity of variance, which are assumptions of the statistical methods used. Normality was assessed using the Shapiro-Wilk test, and homogeneity of variance was assessed using Bartlett’s test. For comparisons between pre- and post-scores in the horizontal ladder test, a paired-sample t-test was applied. For comparisons among three groups in histological evaluations, behavioral tests, and in vitro experiments, a one-way ANOVA followed by Holm–Šídák’s multiple comparisons test was used when the assumptions of normality and equal variance were met. When normality or homogeneity of variance was not present, the Mann–Whitney U test was used for pairwise comparisons. The resulting p-values were adjusted using Holm’s method to account for multiple comparisons. In addition to p-values, effect sizes (Cohen’s d) were calculated to indicate the magnitude of the differences. A p-value of less than 0.05 was considered statistically significant. All quantitative data are presented as the mean values with SDs.

To minimize potential assessment bias, all animals were assigned unique identification numbers that were used throughout the study. The evaluators conducting cell counting and behavioral assessments were blinded to the group assignments. The data were collected and analyzed without knowledge of the treatment groups.

The minimum sample size for the behavioral test was calculated based on preliminary experiments to achieve 80% power with 5% alpha error, assuming a mean difference of 0.3 and an SD of 0.25 on the horizontal ladder test as the primary endpoint. The total sample size was calculated to be *n* = 33 in this case. In previous studies [49, 50], a few rats died, particularly during the model creation process, and some model rats did not develop motor impairments. Furthermore, prior to SHED administration, horizontal ladder test scores had to be evenly allocated. Therefore, the number of rats was set to 12 per group for behavioral tests. To allow for these exclusions and to secure an adequate number of animals, a total of 90 neonatal pups were reared. One pup was excluded because of severe underdevelopment, while the remaining 89 pups were included in the experiment. Of these, 65 pups were initially assigned to the HIE group and 24 to the Sham group. During the HIE modeling procedure, 33 pups were excluded because of surgical complications or because they did not develop motor impairment. Moreover, 4 pups were excluded because of a failure in the administration procedure. Considering the total number of SHED cells available and the need to balance the baseline horizontal ladder scores between groups, the number of rats subjected to behavioral tests was as follows: Vehicle, *n* = 20; SHED, *n* = 12; Sham, *n* = 21. After excluding cases with missing data resulting equipment malfunction, the final number of rats included in the analysis was: Vehicle, *n* = 17; SHED, *n* = 9; Sham, *n* = 15 for the horizontal ladder and cylinder tests, and Vehicle, *n* = 12; SHED, *n* = 10; Sham, *n* = 13 for the shuttle avoidance test.

## Results

### SHED ameliorates motor and memory learning deficits in cerebral palsy models with neurological symptoms

**Fig. 1:**
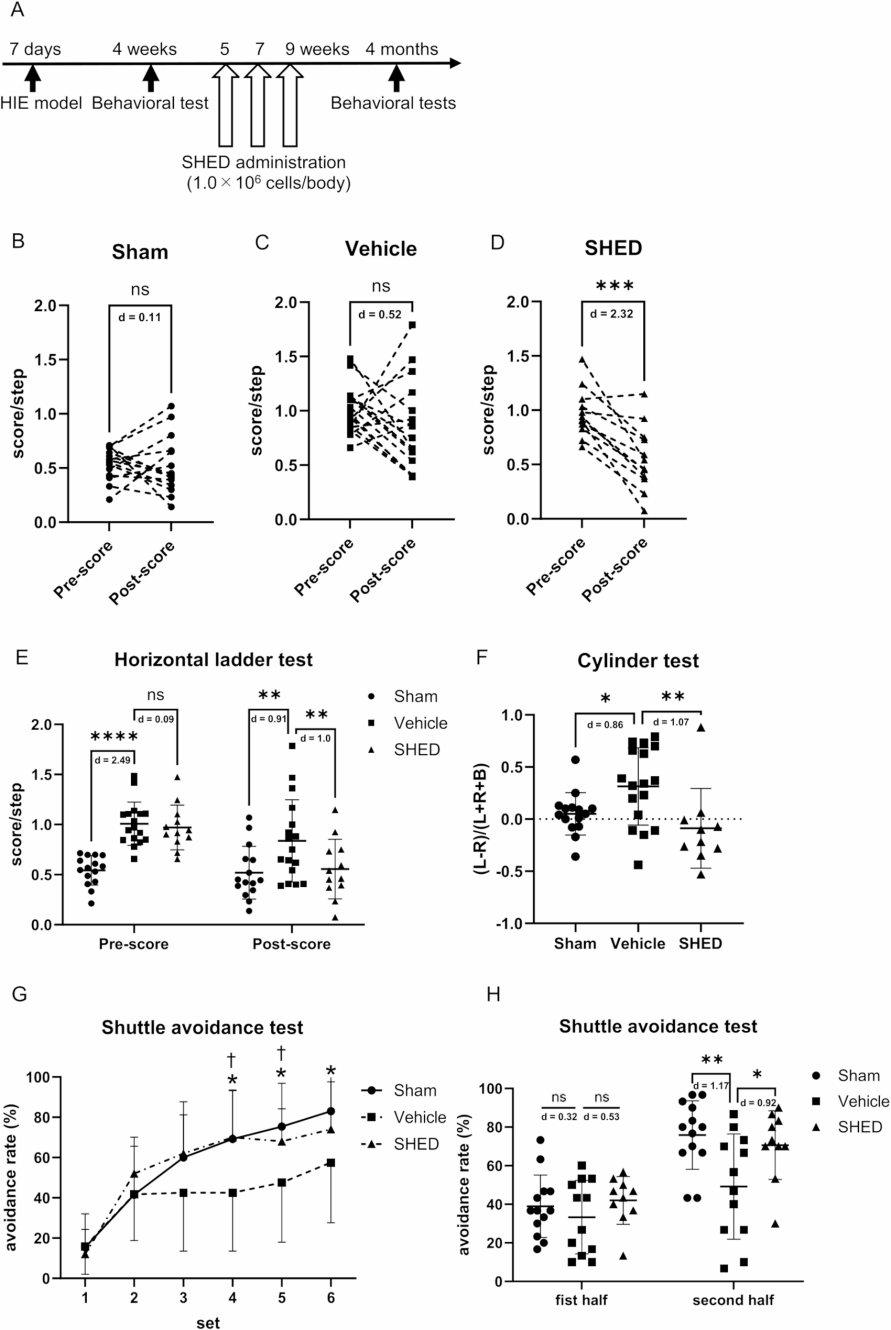
Effect of SHED administration on motor and memory learning deficits using a cerebral palsy model. **A** Timeline of SHED administration and behavioral tests in a cerebral palsy model.** B**–**D** Horizontal ladder test scores for each rat before and after treatment. Higher scores indicate more severe motor impairment.** E** The average scores for the horizontal ladder test before and after administration in each group.** F** The average scores for the cylinder test in each group. The average of the preference for the left (ipsilateral) forepaw was calculated.** G** The average scores for the shuttle avoidance test per set in each group. Each rat underwent six sets of 10 sessions. The avoidance rate was directly proportional to the memory learning ability.** H** The average scores for the shuttle avoidance test in the first half (1–3 sets) and second half (4–6 sets) for each group. Data are presented as the mean ± SD; Vehicle: *n* = 17, SHED: *n* = 12, Sham: *n* = 15 (horizontal ladder test, cylinder test), Vehicle: *n* = 12, SHED: *n* = 10, Sham: *n* = 13 (shuttle avoidance test); * *p* < 0.05, ** *p* < 0.01, *** *p* < 0.001, **** *p* < 0.0001; the paired t-test (**B**–**D**), one-way ANOVA with Holm–Šídák’s multiple comparisons test (E, H) or the Mann–Whitney U test with Holm’s adjustment for multiple comparisons (**F**). * *p* < 0.05 (Sham vs. Vehicle), † *p* < 0.05 (Vehicle vs. SHED); the Mann–Whitney U test with Holm’s adjustment for multiple comparisons (**G**). Values of Cohen’s d are indicated in the graph to represent the effect sizes of group differences

To assess whether SHED administration to cerebral palsy models with neurological symptoms ameliorated those symptoms, we subjected 7-days-old rats to hypoxic-ischemic insults and performed the horizontal ladder test when they were 4 weeks old. Rats with significantly worse scores in this behavioral test were given three intravenous injections of SHED (Fig. 1A). The score on the horizontal ladder test at 4 months of age was significantly decreased only after SHED treatment (Fig. 1B–E). When the cylinder test was performed on animals postadministration, forelimb use preference was significantly decreased in the SHED group compared with the vehicle group (Fig. 1F). Furthermore, when the shuttle avoidance test was performed, the avoidance rate in the second half was significantly higher in the SHED group compared with the vehicle group (Fig. 1G, H).

### Quantum dot-labeled SHEDs migrate to the brain after intravenous administration

**Fig. 2:**
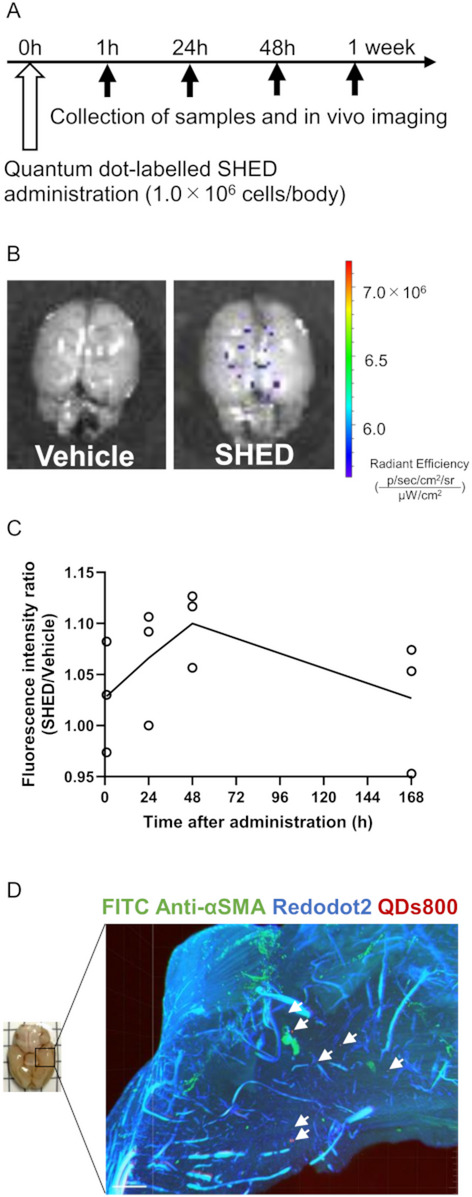
Quantum dot-labeled SHED migrates to the brain following intravenous administration. **A** Timeline of quantum dot-labeled SHED administration and sample collection for in vivo imaging.** B** Representative images of the brain 24 h following the administration of quantum dot-labeled SHED and the vehicle. Increased fluorescence intensity was observed only in brains administered quantum dot-labeled SHED.** C** The mean fluorescence intensity ratio at each time point. Fluorescence intensity ratios were calculated as follows: fluorescence intensity of the brain following quantum dot-labeled SHED administration/mean fluorescence intensity of the brain after vehicle administration.** D** 3D image of the injured cortex 24 h following quantum dot-labeled SHED administration. White arrowheads indicate quantum dot-labeled SHED. Bar = 700 μm. Data are presented as mean values; Vehicle: *n* = 3, SHED: *n* = 3, at each time point

To assess the migration and biodistribution of intravenously administered SHED, we administered QDs-labeled SHED intravenously to cerebral palsy model and measured the fluorescence intensity in the brain at several time points (Fig. 2A). An increased fluorescence intensity was observed only in brains administered with QDs-labeled SHED (Fig. 2B). The fluorescence intensity ratio, corrected for background in the vehicle group, increased transiently 24–48 h after QDs-labeled SHED administration (Fig. 2C). Furthermore, 3D images of these brain tissues showed multiple quantum dot fluorescence in the cortex (Fig. 2D).

### Administration of SHED to rat models of cerebral palsy enhances neurogenesis

**Fig. 3:**
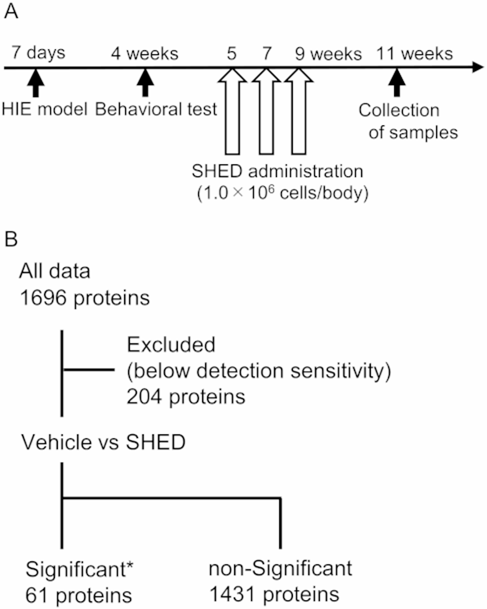
Proteomic analysis of the brain following SHED administration.** A** Timeline of SHED treatment and sample collection for proteomic analyses.** B** Flowchart of the comprehensively analyzed proteins. The expression of 61 proteins was significantly different between the SHED and vehicle groups. Vehicle: *n* = 6, SHED: *n* = 6; * *p* < 0.05 (Student`s t-test) and FDR < 0.1 (Storey method) or the protein expression of all samples in one group is below detection sensitivity

To elucidate the mechanism of the ameliorated neurological symptoms in the cerebral palsy model, we administered SHED to the cerebral palsy model and collected brain samples 2 weeks after the last SHED administration (Fig. 3A). Comprehensive analysis of proteins extracted from the brain samples showed that 61 proteins differed significantly between the SHED and vehicle groups (Fig. 3B). GO analyses were performed on these proteins and five clusters were extracted that met an enrichment score of > 2.0. The enrichment score of the cluster related to neurogenesis was the highest among these clusters (Table 1).

**Table 1:** Clusters with an enrichment score of > 2.0 (Gene ontology analysis)

Cluster	Enrichment Score	Term-name	*p*-value
Neurogenesis	3.30	Regulation of neuron projection development	3.2 × 10⁻⁵
		Neuron projection development	4.0 × 10⁻⁵
		Regulation of cell projection organization	6.2 × 10⁻⁵
		Regulation of nervous system development	1.9 × 10⁻⁴
		Neuron development	2.2 × 10⁻⁴
		Regulation of neuron differentiation	2.3 × 10⁻⁴
		Regulation of neurogenesis	2.8 × 10⁻⁴
		Cell projection organization	8.4 × 10⁻⁴
		Neurogenesis	9.8 × 10⁻⁴
		Regulation of cell development	1.1 × 10⁻³
		Neuron generation	1.4 × 10⁻³
		Neuron differentiation	1.5 × 10⁻³
		Regulation of cell differentiation	1.2 × 10⁻²
		Cell development	1.3 × 10⁻²
Cell morphogenesis	2.70	Regulation of cell morphogenesis	3.2 × 10⁻⁴
		Cell morphogenesis	2.9 × 10⁻³
		Regulation of anatomical structure morphogenesis	3.0 × 10⁻³
		Cellular component morphogenesis	5.8 × 10⁻³
Positive regulation of neurogenesis	2.68	Positive regulation of neuron projection development	2.1 × 10⁻⁴
		Positive regulation of neurogenesis	6.6 × 10⁻⁴
		Positive regulation of neuron differentiation	8.7 × 10⁻⁴
		Positive regulation of cell projection organization	9.9 × 10⁻⁴
		Positive regulation of nervous system development	1.4 × 10⁻³
		Positive regulation of cell development	1.8 × 10⁻³
		Positive regulation of cell differentiation	3.5 × 10⁻²
		Positive regulation of the developmental process	3.6 × 10⁻²
Axonogenesis	2.34	Regulation of axonogenesis	6.3 × 10⁻⁴
		Neuron projection morphogenesis	1.6 × 10⁻³
		Cell projection morphogenesis	1.9 × 10⁻³
		Cell part morphogenesis	2.3 × 10⁻³
		Axonogenesis	4.7 × 10⁻³
		Regulation of cell morphogenesis involved in differentiation	6.6 × 10⁻³
		Axon development	7.8 × 10⁻³
		Cell morphogenesis involved in neuron differentiation	1.7 × 10⁻²
		Cell morphogenesis involved in differentiation	5.2 × 10⁻²
Phosphorylation	2.14	Regulation of the phosphate metabolic process	5.1 × 10⁻³
		Regulation of the phosphorus metabolic process	5.3 × 10⁻³
		Regulation of phosphorylation	5.5 × 10⁻³
		Regulation of protein phosphorylation	7.5 × 10⁻³
		Regulation of the protein modification process	1.9 × 10⁻²

**Fig. 4:**
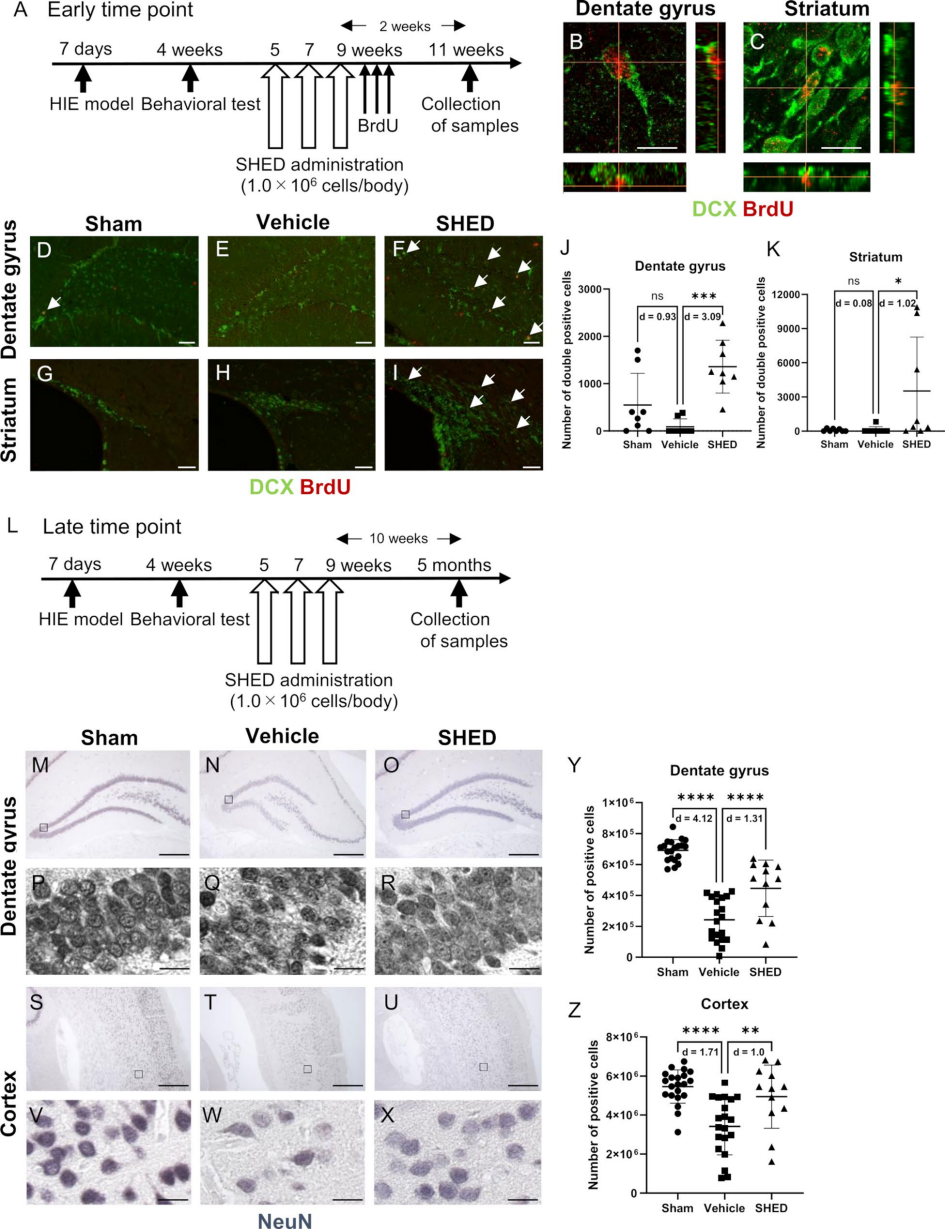
SHED administration enhances neurogenesis in the hippocampal dentate gyrus and subventricular zone. **A** Timeline of SHED administration and sample collection for histological evaluation (early time point).** B**,** C** Representative images of doublecortin (DCX, green) and bromodeoxyuridine (BrdU, red) double-positive cells in the hippocampal dentate gyrus or striatum. Z-stacks were established to verify that they were truly double-positive. Bar = 10 μm.** D**–**K** Representative image of DCX (green) and BrdU (red) immunostaining in the hippocampal dentate gyrus or striatum of each group. The number of DCX/BrdU double-positive cells (white arrow) was increased in the SHED group. Bar = 100 μm.** J**,** K** The average number of DCX/BrdU double-positive cells in the hippocampal dentate gyrus or striatum in each group.** L** Timeline of SHED administration and sample collection for histological evaluation (late time point).** M**–**O** Representative image of NeuN (blue) immunostaining in the hippocampal dentate gyrus of each group. Bar = 500 μm** P**–**R** Representative image of the higher-magnification view of each group. Bar = 20 μm** S**–**U** Representative image of NeuN immunostaining in the cortex of rats of each group. Bar = 500 μm** V**–**X** Representative image of a higher-magnification view of each group. Bar = 20 μm. The number of NeuN-positive cells was increased in the SHED group. **Y**,** Z** Average Number of NeuN-positive cells in the hippocampal dentate gyrus or cortex of each group. Data are presented as the mean ± SD; Vehicle: *n* = 8, SHED: *n* = 8, Sham: *n* = 8 (early time point), Vehicle: *n* = 21, SHED: *n* = 12, Sham: *n* = 20 (late time point); * *p* < 0.05, ** *p* < 0.01, *** *p* < 0.001, **** *p* < 0.0001; the Mann–Whitney U test with Holm’s adjustment for multiple comparisons. Values of Cohen’s d are indicated in the graph to represent the effect sizes of group differences

In addition, a histological evaluation was performed to confirm whether neurogenesis was enhanced after SHED administration. SHED and subsequently BrdU were administered to the cerebral palsy model rats, and brain tissue samples were collected two weeks after the administration (11 weeks of age; the early time point; Fig. 4A). The number of BrdU and DCX double-positive cells in the injured hippocampal dentate gyrus and striatum was significantly higher in the SHED group compared with the vehicle group (Fig. 4B–K). Then, SHED was administered to the cerebral palsy model, and brain tissue was collected 10 weeks after the administration of SHED (5 months of age; the late time point; Fig. 4L). The number of NeuN-positive cells in the hippocampal dentate gyrus and cortex on the injured side was significantly increased in the SHED group compared with the vehicle group (Fig. 4M–Z). To determine whether the increase in mature neurons was the result of enhanced neurogenesis or reduced apoptosis, immunohistochemical staining was conducted for active caspase-3 in the hippocampal dentate gyrus and cortex on the injured side (11 weeks of age; the early time point). The number of active caspase-3–positive cells was not significantly different among the groups in either brain region (Additional File 4: Supplementary Fig. 2).

### SHED has a stronger neurogenic effect compared with other stem cells due to the paracrine effect.

**Fig. 5:**
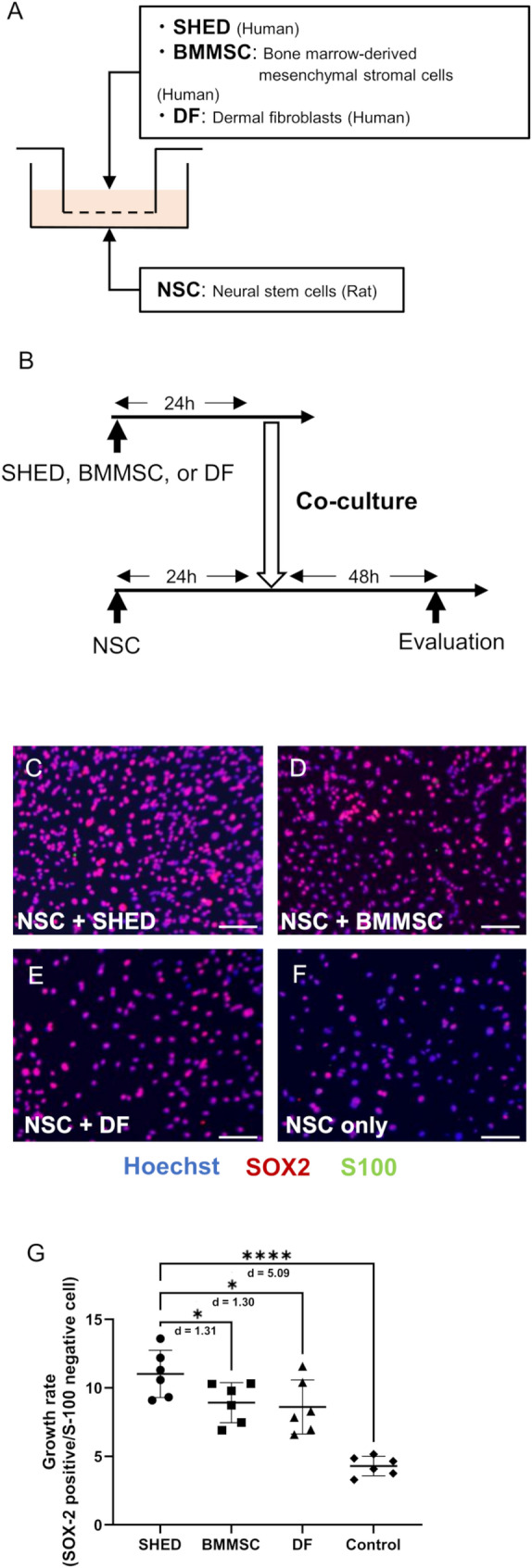
SHED has a stronger neurogenic effect compared with other cells because of the paracrine effect. **A** Schematic diagram of non-contact coculture of NSCs with other stem cells.** B** The timeline of coculture of NSCs with other stem cells.** C**–**F** Representative image of SOX2 (red) and S100 (green) immunostaining of the NSCs following co-culture with each stem cell. Bar = 100 μm.** G** The mean growth rate in each group. The growth rate was higher in the SHED group compared with that in other stem cells. Data are presented as the mean ± SD (*n* = 6), *p < 0.05, ****p < 0.0001; one-way ANOVA with Holm–Šídák’s multiple comparisons test. Values of Cohen’s d are indicated in the graph to represent the effect sizes of group differences

To assess the effect of SHED or other stem cells on the NSC proliferative effect, non-contact co-cultures with NSC were performed (Fig. 5A, B). The NSC growth rate, which was defined as SOX2-positives/S100-negatives 48 h after the beginning of coculture, was highest in the SHED group (Fig. 5C–G). Almost no S100-positive cells could be identified in all groups.

To evaluate the differentiation capacity of NSCs that proliferated following SHED co-culture, we extended the culture period and performed immunostaining on day 14. Most of the cells were positive for MAP2, a marker of mature neurons, and PSD95-positive puncta were observed along the dendrites, which indicates the initiation of synapse formation (Additional File 5: Supplementary Fig. 3).

### RNA-sequence of the co-cultured neural stem cells

**Fig. 6:**
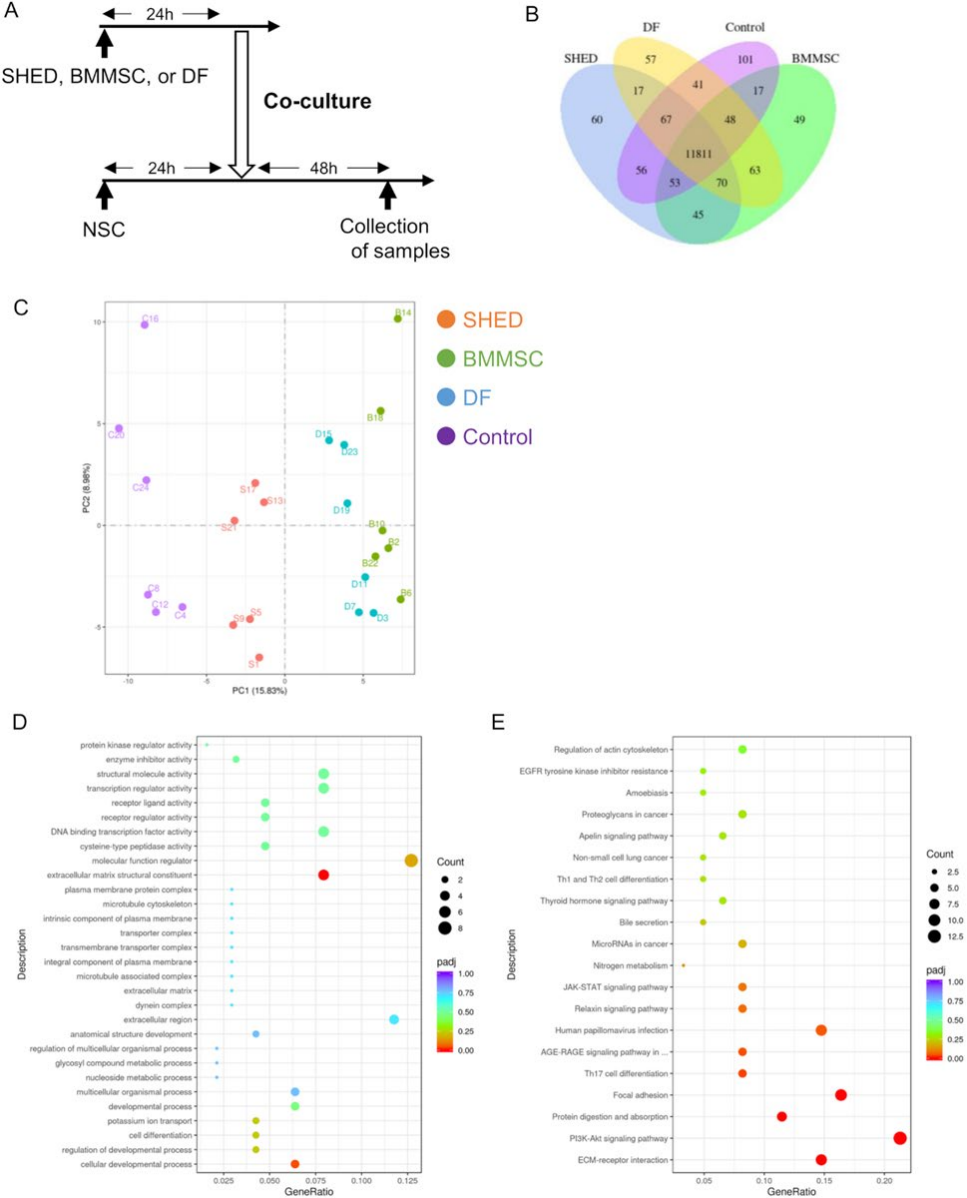
RNA-sequence analysis of cocultured neural stem cells.** A** Timeline for the coculture of NSCs with other stem cells and sample collection for RNA-sequence analysis.** B** Coexpression Venn diagram. Overlapping regions show the number of genes that are coexpressed in at least two groups. Differentially expressed genes were identified in each group.** C** Principal component analysis. The SHED group contained a distinct population from the other groups.** D**,** E** Scatter plot for the Gene Ontology (GO) terms and Kyoto Encyclopedia of Genes and Genomes (KEGG) pathways enriched with differentially expressed genes (DEGs) identified in the SHED vs. control comparison. The point size represents the number of DEGs, whereas the colors indicate the padj ranges. The abscissa is the ratio of the number of DEGs annotated with the GO term or KEGG pathway relative to the total number of DEGs, and the ordinate is the GO term or KEGG pathway. Genes associated with cellular developmental processes and the PI3K-Akt signaling pathway differed significantly between the SHED and control groups

RNA was extracted from the cocultured NSC and RNA-sequense was performed to assess the genes whose expression was altered in the NSCs after co-culturing with the SHED, BMMSC, or DF (Fig. 6A). Specific differentially expressed genes were identified in each group (Fig. 6B), and further principal component analyses showed that the SHED group formed a distinct population from the other groups (Fig. 6C). Then, GO and KEGG pathway analyses were performed using the genes whose expression differed between the SHED and control groups, and several GO terms and KEGG pathways differed significantly between the two groups. The number of differentially expressed genes associated with cellular developmental processes (Fig. 6D) and the PI3K-Akt signaling pathway (Fig. 6E) were the highest among them.

### HGF secreted by SHED enhances neural stem cell proliferation through Akt phosphorylation

**Fig. 7:**
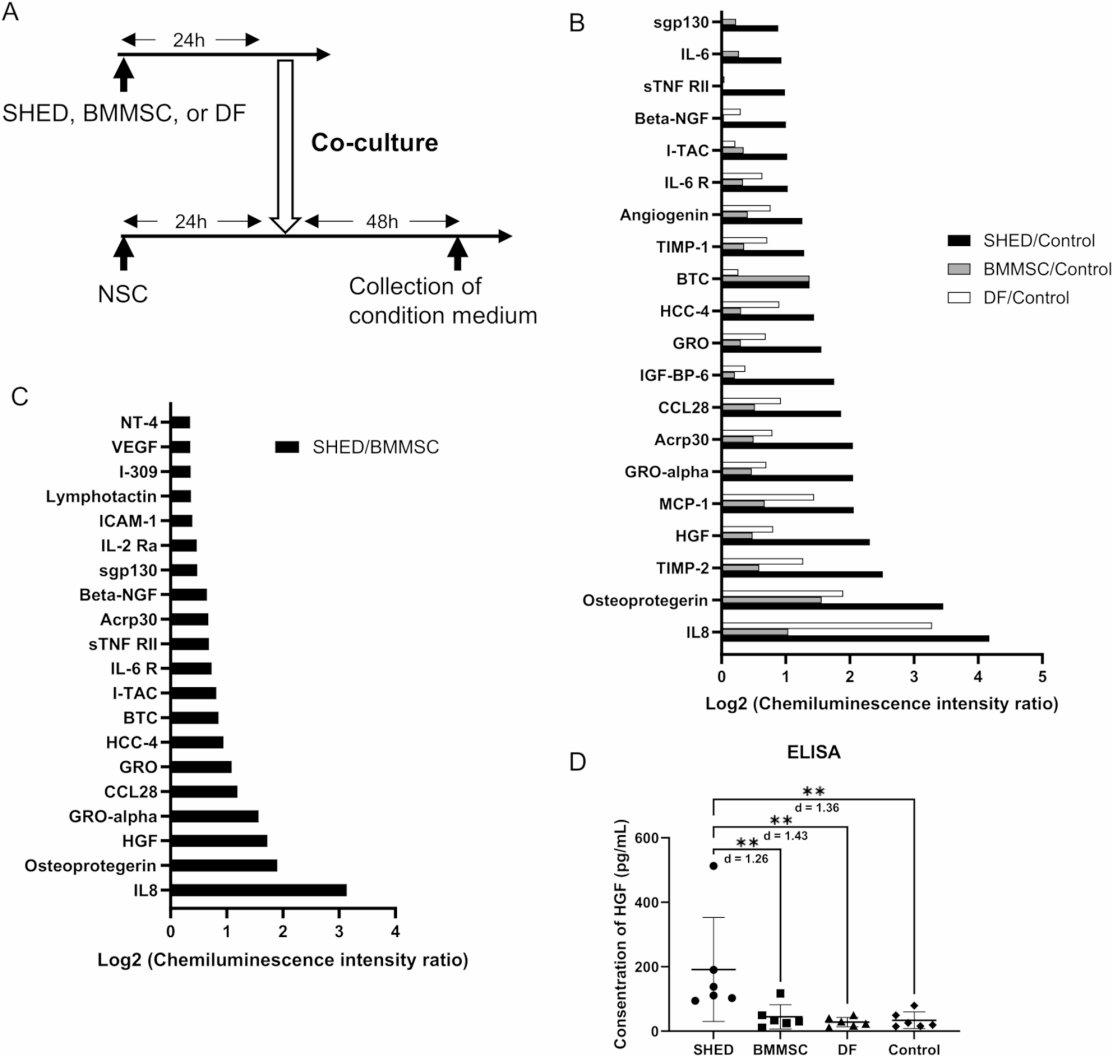
Conditioned medium derived from SHED contains higher HGF levels compared with that from other stem cells.** A** Timeline of the coculture of NSCs with other stem cells and the collection of conditioned medium.** B** Ratio of the chemiluminescence intensity between each group and the Control.** C** Ratio of the chemiluminescence intensity between the SHED and BMMSC groups. The top 20 out of 120 total cytokines with higher values in the SHED group are indicated. The values are presented on a logarithmic scale. The expression of several trophic factors associated with NSC proliferation was higher in the SHED group. HGF was highly expressed in the SHED-conditioned medium compared with other stem cells.** D** The mean concentration of HGF in each conditioned medium was measured using an ELISA kit. Data are presented as the mean ± SD (*n* = 6), **p < 0.01, ***p < 0.001, ****p < 0.0001; the Mann–Whitney U test with Holm’s adjustment for multiple comparisons. Values of Cohen’s d are indicated in the graph to represent the effect sizes of group differences

To evaluate differences in trophic factors secreted by SHED or other stem cells, condition medium samples after coculture were comprehensively assessed using the Human Cytokine Antibody Array (Fig. 7A). As the NSC used for coculture were of rat origin, trophic factors secreted from stem cells of human origin were evaluated. The expression of several trophic factors associated with NSC proliferation was higher in the SHED group (Fig. 7B), and we focused particularly on HGF, which was highly detected in the SHED-conditioned medium compared with BMMSC (Fig. 7C) and related to cellular developmental processes and the PI3K-Akt signaling pathway [44, 46]. The HGF concentration in the conditioned medium determined by ELISA was significantly higher than that in other groups (Fig. 7D).

**Fig. 8:**
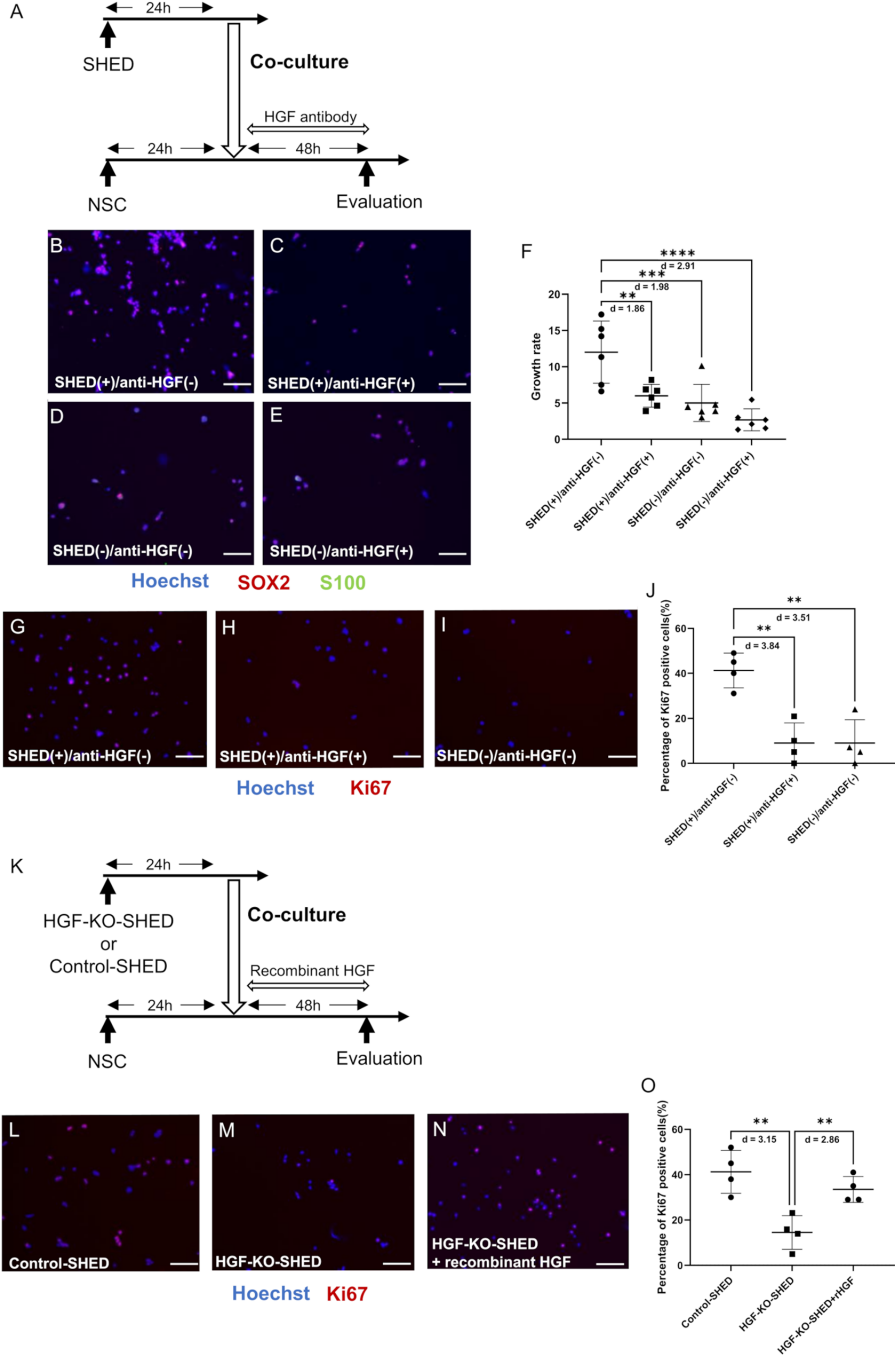
HGF secreted by SHED enhances neural stem cell proliferation. **A** Timeline of the coculture of NSCs with SHED and treatment with HGF-neutralizing antibody.** B**–**E** Representative image of SOX2 (red) and S100 (green) immunostaining in the NSCs after coculture under different conditions (with or without SHED, with or without the neutralizing antibody). Bar = 100 μm.** F** The mean growth rate in each group.** G**–**I** Representative image of Ki67 (red) immunostaining in NSCs after co-culture under different conditions (with or without SHED, with or without the neutralizing antibody). Bar = 100 μm.** J** Average percentage of Ki67-positive cells in each group. NSC proliferation was significantly decreased following the addition of HGF antibody. No significant changes were observed when NSCs were cultured alone or when HGF antibody was added.** K** Timeline of the coculture of NSCs with HGF-KO SHED and the rescue experiment using recombinant HGF.** L**–**N** Representative images of Ki67 (red) immunostaining in NSCs after coculture under various conditions (with Control-SHED, HGF-KO SHED, or HGF-KO SHED containing recombinant HGF). Bar = 100 μm.** O** Average percentage of Ki67-positive cells in each group. NSC proliferation was significantly decreased when co-cultured with HGF-KO SHED. This reduction was rescued by recombinant HGF treatment. Data are presented as the mean ± SD.** F** *n* = 6;** J** and** O**: *n* = 4. **p < 0.01, ***p < 0.001, ****p < 0.0001; the Mann–Whitney U test with Holm’s adjustment for multiple comparisons (**F**) or one-way ANOVA with Holm–Šídák’s multiple comparisons test (**J**,** O**). Values of Cohen’s d are indicated in the graph to represent the effect sizes of group differences

Then, anti-human HGF antibodies were added to the medium at the beginning of the co-culture of SHED and NSC (Fig. 8A). The growth rate of NSC, which was defined as SOX2-positives/S100-negatives at 48 h of the co-culture, was significantly decreased by the addition of HGF antibodies. However, no significant changes in the NSC growth rate were observed when NSCs were cultured alone and HGF antibodies were added (Fig. 8B–F). Consistently, the proportion of Ki67-positive cells, which is another marker of proliferation, was significantly increased in the SHED group and reduced to control levels following HGF antibody treatment (Fig. 8G–J).

NSCs were co-cultured using the same protocol with either HGF-KO SHED or control SHED. Recombinant human HGF was added to the culture medium at the beginning of co-culture (Fig. 8K). The proportion of Ki67-positive cells was significantly decreased in the HGF-KO SHED coculture group compared with that in the control SHED group; however, supplementation with recombinant HGF restored Ki67-positive cell levels to those observed in the control SHED group (Fig. 8L–O).

**Fig. 9:**
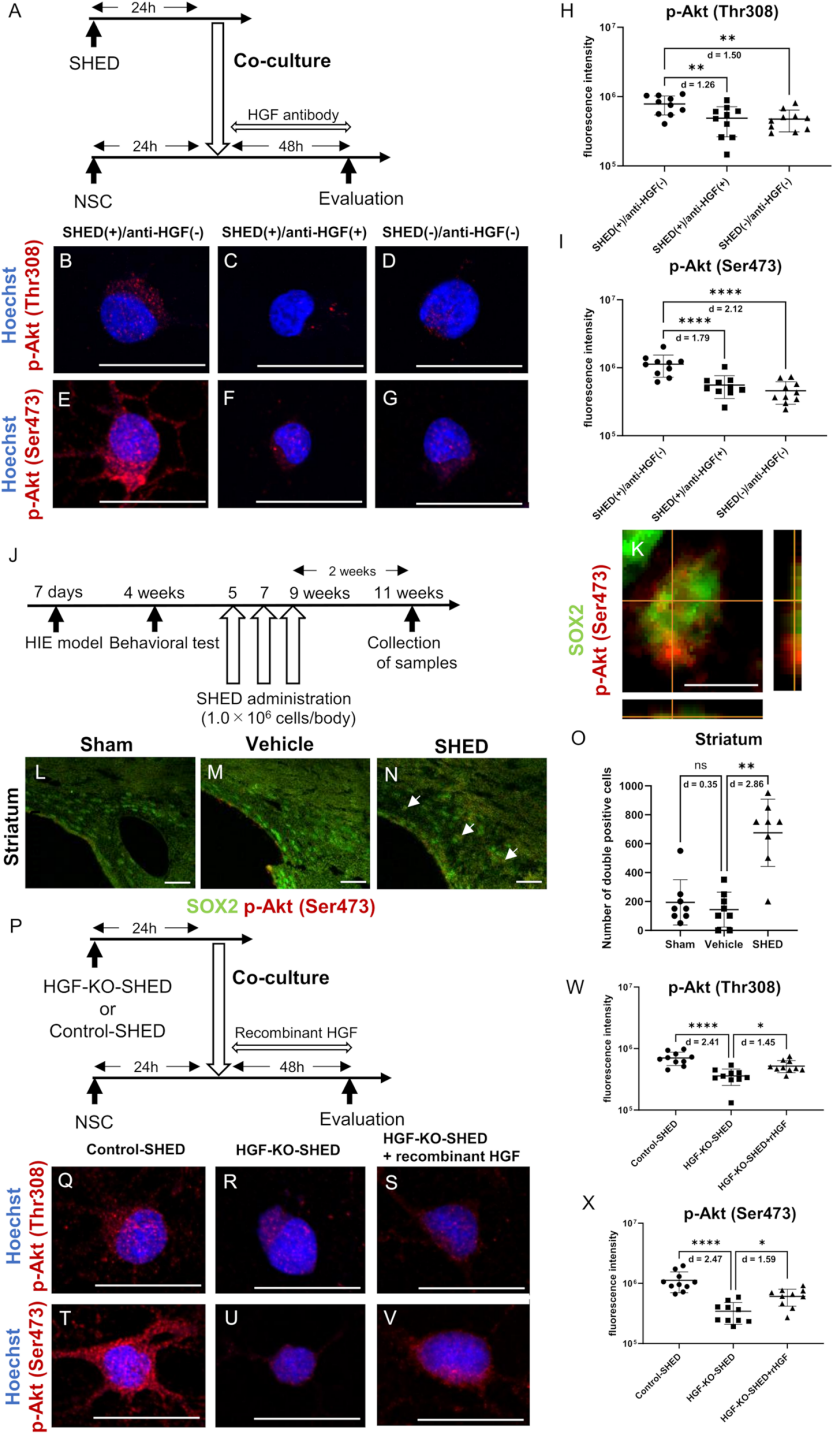
HGF secreted by SHED promotes phosphorylation of Akt in neural stem cells. **A** Timeline for the coculture of NSCs with SHED and the application of HGF-neutralizing antibody.** B**–**G** Representative images of p-Akt immunostaining in NSCs following coculture under different conditions (with or without SHED, with or without neutralizing antibody).** B**–**D** p-Akt (Thr308),** E**–**G** p-Akt (Ser473). Bar = 20 μm.** H**,** I** Average fluorescence intensity of p-Akt (Thr308)** H** and p-Akt (Ser473)** I** in each group.** J** Timeline of SHED administration and sample collection for histological evaluation.** K** Representative images of SOX2 (green) and p-Akt (Ser473, red) double-positive cells in the striatum. Z-stacks were established to verify that they were truly double-positive. Bar = 10 μm.** L**–**N** Representative image of SOX2 (green) and p-Akt (Ser473, red) immunostaining in the striatum of each group. The number of SOX2/p-Akt (Ser473) double-positive cells (white arrow) was increased in the SHED group. Bar = 50 μm.** O** The average number of SOX2/p-Akt (Ser473) double-positive cells in the striatum of each group.** P** Timeline of the coculture of NSCs with HGF-KO SHED and the rescue experiment using recombinant HGF.** Q**–**V** Representative images of p-Akt immunostaining in NSCs following coculture under different conditions (with Control-SHED, HGF-KO SHED, or HGF-KO SHED supplemented with recombinant HGF).** Q**–**S** p-Akt (Thr308),** T**–**V**: p-Akt (Ser473). Bar = 20 μm.** W**,** X** Average fluorescence intensity of p-Akt (Thr308) **W** and p-Akt (Ser473) **X** in each group. Data are presented as the mean ± SD. **H**,** I**,** W**,** S** *n* = 10 per group, measured from three independent wells. **O** *n* = 8 per group. **p < 0.01, ***p < 0.001, ****p < 0.0001; the Mann–Whitney U test with Holm’s adjustment for multiple comparisons** (O)** or one-way ANOVA with Holm–Šídák’s multiple comparisons test (**H**,** I**,** W**,** X**). Values of Cohen’s d are indicated in the graph to represent the effect sizes of group differences

To determine whether the PI3K–Akt signaling pathway was activated downstream of SHED-derived HGF, immunostaining for phosphorylated Akt at Thr308 and Ser473 was done under the same coculture conditions (Fig. 9A). Quantitative analysis revealed that the fluorescence intensities of p-Akt (Thr308) and p-Akt (Ser473) were significantly increased in NSCs form the SHED group. This increase was significantly suppressed by HGF-neutralizing antibody treatment, returning to levels comparable with the control group (Fig. 9B–I).

Histological evaluation was performed to determine whether Akt phosphorylation was enhanced following SHED administration in vivo. Brain tissue samples were collected from cerebral palsy model rats two weeks after SHED treatment (Fig. 9J). The number of p-Akt (Ser473) and SOX2 double-positive cells in the injured striatum was significantly higher in the SHED group compared with that in the vehicle-treated group (Fig. 9K–O). Moreover, NSCs were co-cultured using the same protocol with either HGF-KO SHED or control SHED. Recombinant human HGF was added to the medium at the beginning of coculture (Fig. 9P). The fluorescence intensities of p-Akt (Thr308) and p-Akt (Ser473) were significantly decreased in the HGF-KO SHED co-culture group compared with those in the control SHED group; however, supplementation with recombinant HGF restored the fluorescence intensities of both p-Akt isoforms to levels comparable with those observed in the control SHED group (Fig. 9Q–X).

## Discussion

It is necessary to develop novel therapies for cerebral palsy that are effective even after symptoms are identified in the chronic period. This study demonstrated that SHED ameliorated the motor, memory, and learning impairments in a cerebral palsy model following enhancements in neurogenesis in the adult brain after administration. Furthermore, SHEDs have been shown to have a strong NSC proliferative effect through alterations in the PI3K-Akt signaling pathway via specifically secreted HGF compared with other stem cells.

### SHED is effective even when administered to a cerebral palsy model with neurological symptoms

Several clinical trials have been conducted on stem cell therapy for the chronic stages of perinatal brain injuries. A systematic review conducted in 2022 reported nine randomized controlled trials of stem cell-based therapy for cerebral palsy [15]; Although some preclinical studies, such as those using cord blood-derived mesenchymal stromal cells, have shown promise in HIE model animals at 21 days of age [73], the underlying mechanisms for the therapeutic effects in chronic-phase models remain unclear. Moreover, to the best of our knowledge, there are no reports confirming the improvement in neurological symptoms between before and after stem cell administration in the same subject.

In this study, we defined a cerebral palsy model as an animal with coordinated motor impairment in a behavioral test after hypoxic-ischemic insult. We observed ameliorations in coordinated motor impairment in the same animal even when SHED was administered at 35 days of age in rats, which corresponds to pre-adolescence [53] in humans. Furthermore, this cerebral palsy model also demonstrated improvements in memory and learning impairment after the administration of SHED, indicating that SHED is useful as a novel treatment option for cerebral palsy, with potential therapeutic effects even after symptoms are identified in the chronic phase. These effects may be attributed to the unique trophic factor profile of SHED, including HGF, as well as their superior neurogenic potential compared with other stem cell types.

### SHED enhances the proliferation and differentiation of neural stem cells in the adult brain through HGF-mediated Akt phosphorylation

Noncontact co-cultures of NSC isolated from the hippocampal dentate gyrus of adult rats with SHED or other stem cells showed that NSC growth was most enhanced when co-cultured with SHED (Fig. 6G). In adult brains, endogenous NSCs and their neurogenesis are observed in the SVZ of the lateral ventricles and in the granular cell layer of the hippocampal dentate gyrus [74]. In addition, neural stem or progenitor cells in the SVZ increase after a cerebral ischemic insult and migrate toward the injured striatum using blood vessels as scaffolds [75, 76], some of which differentiated into mature neurons in the striatum [77]. These phenomena are considered some of the mechanisms underlying the amelioration of motor funciton after cerebral ischemic insult [78]. This increment in neurogenesis and migration to the injury site peaks reduces approximately two weeks post-injury [77, 78]. In this study, brain tissue samples were collected two weeks after the last administration of SHED, which corresponds to 10 weeks after the hypoxic-ischemic insult. By this time, the process of enhanced endogenous NSC proliferation after a cerebral ischemic insult has already passed its peak. Because BrdU was administered immediately after SHED administration, BrdU and DCX double-positive cells are those that proliferated and differentiated into neurons immediately after SHED administration, suggesting that treatment with SHED re-activated the proliferation of endogenous NSCs and their differentiation into neurons that have already passed their peak.

This phenomenon was also observed in the hippocampal dentate gyrus, where the number of mature neurons in the granular cell layer increased in the late phase after 10 weeks of SHED treatment, indicating that the effect induced by SHED treatment lasted until the chronic phase. To determine whether this increase was the result of enhanced neurogenesis or reduced apoptosis, active caspase-3 staining was performed. No significant differences were observed among the SHED, vehicle, and sham groups. This suggests that the increase in mature neurons was primarily attributed to enhanced neurogenesis rather than decreased apoptosis. The suppression of neurogenesis in the granular cell layer of the adult brain is associated with learning impairment [79], indicating that the improvement in neurogenesis in the granular cell layer and the increase in the number of mature neurons by SHED administration may contribute to the amelioration of learning impairment observed in the shuttle avoidance behavioral test in this study. These results indicate that SHED is a promising novel treatment option for cerebral palsy.

To further examine the mechanism underlying this SHED-induced neurogenic effect, we focused on the profile of SHED-secreted trophic factors. A comprehensive comparison of conditioned medium from SHED, BMMSC, and DF revealed distinct expression profiles (Fig. 7B). Of the factors implicated in neurogenesis, we focused on HGF, which was enriched in SHED-conditioned medium and is linked to developmental processes and the PI3K–Akt signaling pathway. The role of HGF was confirmed through functional assays. NSC proliferation decreased when HGF-neutralizing antibodies were added to SHED–NSC co-culture (Fig. 8B–J) or when HGF-KO SHED were used. This reduction was rescued by recombinant HGF (Fig. 8L–O). These observations indicate that HGF is a key mediator of the SHED-induced neurogenic effect. Consistent with our findings, Nicoleau et al. found that intraventricular injection of HGF in adult mice increased cell proliferation in the SVZ, whereas injection of HGF-neutralizing antibodies caused a decrease [71]. This supports a role for HGF in inducing NSC proliferation.

Met is the receptor for HGF and is expressed throughout the nervous system during development and adulthood. It supports neuronal growth and survival [46]. Binding of HGF to Met activates multiple downstream cascades, including PI3K. Akt, a principal effector of the PI3K pathway, and promotes cell proliferation, survival, and metabolism. It requires phosphorylation at Thr308 by PDK1 and at Ser473 by mTORC2 for full activation [44]. Coculture with SHED significantly enhanced Akt phosphorylation in NSCs, and this effect was attenuated by HGF neutralization (Fig. 9B–I) or by coculture with HGF-KO SHED, which was restored by recombinant HGF treatment (Fig. 9Q–X). HGF stimulates mTORC2 activity, thereby promoting Ser473 phosphorylation of Akt to achieve maximal activation [45]. Torroglosa et al. demonstrated that enhanced Akt phosphorylation promotes NSC proliferation in the SVZ by preventing the nuclear translocation of the cyclin-dependent kinase inhibitor p27 Kip1, thus sustaining cell cycle progression [47]. Taken together, these findings indicate that SHED-derived HGF promotes neural stem-cell proliferation primarily by enhancing Akt phosphorylation, thus delineating a key mechanism for the SHED-induced neurogenic effect.

## Limitations and perspectives

In this study, we demonstrated that SHED ameliorates the motor, memory, and learning impairments observed in a cerebral palsy model. This was accompanied by enhanced neurogenesis in the adult brain. Nonetheless, several limitations should be acknowledged. For example, we used a xenogeneic transplantation model to evaluate the therapeutic effects of human-derived SHED in vivo. Although this model is widely used to assess human cell function, it does not fully replicate the physiological environment of autologous or allogeneic transplantation in humans. The species-specific differences in immune responses, cell–cell interactions, and tissue integration may influence outcomes. Thus, the direct translation of these results to those of clinical studies should be considered cautiously. Future studies using humanized or allogeneic models are essential to validate and extend these results. Moreover, because the structure of SVZ in rodents differs from that in humans [80], it is unclear whether SHED ameliorates neurological symptoms through the same mechanisms that occur in human subjects. However, because increased cell proliferation has been observed in ipsilateral SVZ following ischemic stroke in humans [81], a similar response may be expected in human HIE, which is also a form of ischemic injury. Therefore, caution should be exercised when directly translating these results to those of clinical studies.

Another limitation is the unequal sample sizes used for the experimental groups, particularly for the behavioral assessments. This disparity arose from a combination of biological and technical factors, including unexpected mortality during HIE model induction, the absence of motor impairment in certain animals, and efforts to balance baseline behavioral scores before treatment. To address this issue and reduce potential bias, we performed tests for normality and homogeneity of variance and used appropriate statistical analyses, including nonparametric methods. In addition, effect sizes were reported to facilitate the interpretation of group differences independent of sample size.

Although the sample sizes in our RNA-sequence and proteomic analyses were limited, this design is consistent with common practices in omics research under practical constraints, such as sample availability and cost. Large-scale benchmarking studies [82] have indicated that datasets with as few as three biological replicates can reliably detect markedly differentially expressed genes, which are often of great biological relevance.

The availability of SHED is inherently limited, as they can only be collected within a specific developmental window and from a restricted number of donors. Nevertheless, SHED exhibit superior proliferative capacity compared with other stem cell types [83], which may help to partially overcome the limitations in supply. Despite this advantage, the clinical application of SHED-based products will inevitably involve cells derived from multiple donors, which introduces concerns regarding batch-to-batch variability and quality control. To address these challenges, it may be necessary to define key factors identified in the present study, such as HGF, as Critical Quality Attributes (CQAs). The establishment of CQAs will support standardization efforts and ensure the consistency of SHED-derived cell products.

Although HGF has beneficial roles in neuroprotection and tissue regeneration, it also has well-documented pro-oncogenic attributes, including the promotion of cell proliferation, migration, and angiogenesis [84]. These characteristics raise safety concerns. Therefore, future preclinical studies should include comprehensive safety assessments, such as the evaluation of tumorigenic potential, as part of the translational development of SHED-based therapies.

The SHED dose used in this study was based on previous reports demonstrating efficacy in the acute HIE model [39]. As the expected therapeutic effect was different, it is necessary to explore dosages required for optimal therapeutic effects.

In this study, we determined the effects of SHED up to four months following administration, which corresponds to five months of age in rats. This reflects early adulthood and is considered a long-term period in preclinical models of perinatal brain injury, such as cerebral palsy [53]. Previous studies assessing stem cell efficacy in chronic-phase models have limited their follow-up to approximately 9 weeks of age [73]. Our extended evaluation provides valuable insight into the sustained therapeutic effects of SHED, including functional recovery and enhanced neurogenesis during the chronic phase.

It was also demonstrated that SHED has a proliferative effect on NSCs when co-cultured. However, its effect on the differentiation of proliferated NSCs could not be assessed in the coculture experiments conducted in this study. Indeed, a histological evaluation showed an increase in the number of cells that differentiated into immature neurons 2 weeks after SHED treatment (Fig. 4B–I), which suggests that SHED enhanced the differentiation of proliferating NSCs into neurons. To further examine this phenomenon, we performed an additional experiment using an extended culture period. Two weeks after SHED co-culture, the majority of NSCs expressed MAP2, a marker of mature neurons, and PSD95-positive puncta were observed along dendrites, which indicates synapse formation (Additional File 5: Supplementary Fig. 3). These results suggest that NSCs expanded by SHED retain the potential to differentiate into mature neurons and initiate synaptic connectivity during subsequent maturation.

## Conclusions

This study revealed that SHED administered in the chronic phase ameliorated motor, memory, and learning impairments in a cerebral palsy rat model. Those improvements were accompanied by enhancements in NSC proliferation and differentiation in the adult brain, likely mediated by HGF secretion and activation of the PI3K–Akt signaling pathway. Those results suggest that intravenous SHED has potential for the treatment of children with cerebral palsy.

## Supplementary Information

Below is the link to the electronic supplementary material.

Supplementary Material 1.

Supplementary Material 2.

Supplementary Material 3.

Supplementary Material 4.

Supplementary Material 5.

## Data Availability

All data are available from the corresponding author upon reasonable request. All additional files supporting the findings of this study, including proteomic analysis data and RNA sequencing data, are included in the manuscript as supplementary materials.

## Abbreviations

BMMSC: Bone Marrow-Derived Mesenchymal Stem Cell
BrdU: Bromodeoxyuridine
DCX: Doublecortin
DF: Dermal Fibroblast
ELISA: Enzyme-Linked Immunosorbent Assay
FDR: False Discovery Rate
FPKM: Fragments Per Kilobase of Transcript Per Million Mapped Reads
GO: Gene Ontology
HGF: Hepatocyte Growth Factor
HIE: Hypoxic-ischemic Encephalopathy
LC/MS/MS: Liquid Chromatography/Tandem Mass Spectrometry
NeuN: Neuronal Nuclear Protein
NSC: Neural Stem Cell
PCR: Polymerase Chain Reaction
PFA: Paraformaldehyde
PBS: Phosphate Buffered Saline
QD: Quantum Dot
RI: Refractive Index
SD: Standard Deviation
SHED: Stem Cells from Human Exfoliated Deciduous Teeth
SOX2: SRY-Box Transcription Factor 2
SVZ: Subventricular Zone
