# Supplementary material for: Novel stem cell therapy for cerebral palsy using stem cells from human exfoliated deciduous teeth
Source: Stem Cell Res Ther. 2026 Jan 23;17:44. doi: 10.1186/s13287-025-04828-y (PMC12833939; doi:10.1186/s13287-025-04828-y)
Supplement: Supplementary file 1 — Supplementary Material 1. [file 13287_2025_4828_MOESM1_ESM.docx]

Additional File 1: SUPPLEMENTAL MATERIAL

Novel Stem Cell Therapy for Cerebral Palsy Using Stem cells from human exfoliated deciduous teeth

Takahiro Kanzawa MD^1,2)^, Atsuto Onoda PhD^3)^, Azusa Okamoto^1)^, Xu Yue MD^1,2)^, Ryoko Shimode MD^1,2)^, Yukina Takamoto MD^1,2)^, Sakiko Suzuki MD^1,2)^, Kazuto Ueda MD PhD^1)^, Ryosuke Miura MD^1)^, Toshihiko Suzuki MD PhD^1)^, Naoki Tajiri PT PhD^4)^, Shinobu Shimizu PhD^5)^, Saho Morita^6)^, Hiroshi Yukawa PhD^7,8)^, Hiroshi Kohara^9)^, Noritaka Fukuda^9)^, Yasuyuki Mitani PhD^9)^, Hideki Hida MD PhD^4)^, Yoshiyuki Takahashi MD PhD^2)^, Yoshiaki Sato MD PhD^1)^

Affiliations:

1. Division of Neonatology, Center for Maternal-Neonatal Care, Nagoya University Hospital
   65 Tsurumai-cho, Showa-ku, Nagoya, Aichi 466-8560, Japan
2. Department of Pediatrics, Nagoya University Graduate School of Medicine, Japan
   65 Tsurumai-cho, Showa-ku, Nagoya, Aichi 466-8560, Japan
3. Department of Toxicology and Health Science, Faculty of Pharmaceutical Sciences, Sanyo-Onoda City University
   Daigakudori 1-1-1, Sanyo-Onoda Yamaguchi 756-0884, Japan
4. Department of Neurophysiology and Brain Science, Nagoya City University Graduate School of Medical Science, Japan
   1 Kawasumi, Mizuho-cho, Mizuho-ku Nagoya 467-8601, Japan
5. Department of Advanced Medicine Nagoya University Hospital, Japan
   65 Tsurumai-cho, Showa-ku, Nagoya, Aichi 466-8560, Japan
6. Department of Biomolecular Engineering, Graduate School of Engineering, Nagoya University, Japan
   Furo-cho, Chikusa-ku, Nagoya, 464-8601, Japan
7. Research Institute for Quantum and Chemical Innovation, Institutes of Innovation for Future Society, Nagoya University, Japan
   Furo-cho, Chikusa-ku, Nagoya, 464-8601, Japan
8. Quantum Regenerative and Biomedical Engineering Team, Institute for Quantum Life Science, National Institutes for Quantum Science and Technology, Japan
   4-9-1 Anagawa, Inage-ku, Chiba 263-8555
9. S-Quatre Corporation, Tokyo, Japan

3-8-3 Nihombashihoncho, Chuo-ku, Tokyo 103-0023

Corresponding author: Yoshiaki Sato MD PhD
Corresponding author’s address: 65 Tsurumai-cho, Showa-ku, Nagoya, Aichi 466-8560, Japan
Phone: (+81) 52-744-2294; Fax: (+81) 52-744-2974
Corresponding author’s e-mail address: y.sato@nagoya-u.jp

**Supplemental Methods**

**Generation and Characterization of HGF-Knockout SHED and the Analysis of Secreted Growth Factors**

The cDNA encoding AncBE4max [1] and the corresponding sgRNAs were synthesized by GenScript (Piscataway, NJ, USA). Messenger RNA was isolated using the mMESSAGE mMACHINE kit (Takara Bio, Shiga, Japan) and encapsulated in lipid nanoparticles as previously described [2] to yield hepatocyte growth factor–knockout SHED (HGF-KO-SHED). The sgRNA sequences were as follows: HGF-sgRNA (5’-GGTCACCCACATGGTGCTGC-3’-) and Negative-control sgRNA (5’-AAATGTGAGATCAGAGTAAT-3-‘). Knockout efficiency was assessed by Sanger sequencing using the following primers: HGF-forward (5’-ACTCCGAACAGGATTCTTTC-3’-) and HGF-reverse (5’-GACTAGGTGATGTGGAGTAGTGC-3’).

HGF-KO-SHED were cultured under the same conditions as wild-type SHED. After expansion, the cells were seeded at 6 × 10⁴ cells/cm² and cultured for 24 h, followed by medium replacement with phenol red-free MEM α containing nucleosides (Thermo Fisher Scientific, Waltham, MA, USA). After 48 h of culture, the conditioned medium was collected, centrifuged at 400 × g for 5 min at 4°C, and stored at −80°C until use. The concentration of the secreted soluble factors was measured using the LEGENDplex™ Human Growth Factor Panel (BioLegend, San Diego, CA, USA) based on the manufacturer’s instructions.

**Assessment of Apoptosis Following SHED Administration in the Cerebral Palsy Model**

Brain tissue was collected 2 weeks after the final administration of SHED to the cerebral palsy model rats. Immunohistological staining was performed as previously described [3]. All of the rats were administered three types of mixed anesthetic agents (medetomidine, midazolam, and butorphanol) intraperitoneally. Perfusion fixation was conducted using 4% paraformaldehyde, and serial coronal sections were prepared. After antigen removal, the sections were incubated overnight at 4°C with primary antibody (rabbit anti-active caspase-3; 1:200, BD Biosciences, San Jose, CA, USA) in PBS containing 4% donkey serum (Jackson Immuno Research Laboratories, Baltimore Pike, PA, USA) and 0.1% Triton X-100. The sections were subsequently incubated with the biotinylated secondary antibody (Donkey Anti-Rabbit IgG; Jackson ImmunoResearch) for 60 min, followed by 3% H_2_O_2_ for 10 min. Binding was visualized using a Vectastain ABC kit (Vector Laboratories, Burlingame, CA, USA) with peroxidase detection for 12 min (0.12 mg/ml 3,3’-diaminobenzidine, 0.01% H_2_O_2_, and 0.04% NiCl_2_).

Immunohistochemical images were captured using an inverted fluorescence microscope (IX83, Olympus Corporation, Tokyo, Japan) equipped with a DP73 camera (Olympus Corporation). Active caspase-3 was imaged at low magnification (20× objective lens) and high magnification (40× objective lens).

Sections were prepared at 5 μm and stained at 50-section intervals to count cells that were positive by immunostaining. Caspase-3-positive cells were counted throughout the hippocampal dentate gyrus and cortex using the Stereo Investigator^®^ version 10 stereology software (MicroBrightField Europe EK, Magdeburg, Germany [3]). Quantitation was done using the optical fractionator probe. A 25 μm × 25 μm counting frame was applied to an 80 μm × 190 μm sampling grid in the hippocampal dentate gyrus. In the cortex, a 50 μm × 50 μm counting frame was applied to an 800 μm × 800 μm sampling grid. The optical dissector height was set to 5 μm, with no guard zones at the top or bottom of each section. Positive cells were counted using a 40× objective lens, and Gundersen coefficients of error (m = 1) were ≤0.10 for all estimates.

**Assessment of NSC Differentiation and Functional Network Integration following SHED Coculture**

Adult Rat Hippocampal Neural stem cells (NSCs, SCR022, Merck) were seeded on plates coated with Poly-L-ornithine (P3655, Merck) and Laminin (CC095, Merck), and cultured in DMEM/F12 (Thermo Fisher Scientific) containing 2% B-27 supplement (Thermo Fisher Scientific), 1% GlutaMAX^TM^ supplement (Thermo Fisher Scientific), and 20 ng/mL fibroblast growth factor basic protein (GF003, Merck). The initial seeding density of the NSCs was 1 × 10^4^ cells per well. SHED were seeded onto Transwell inserts (Falcon™ Cell Culture Inserts for 24-well plates with a 0.4 µm PET transparent membrane, Corning, NY, USA). The cells were cultured in MesenCult™-hPL Medium (STEMCELL Technologies, Vancouver, Canada) containing 1% GlutaMAX^TM^ supplement (Thermo Fisher Scientific, Waltham, MA, USA). After 24 h, the medium was changed to MEM α containing nucleosides, but no phenol red (Thermo Fisher Scientific). The inserts were transferred to wells containing NSCs. In the control group, only the inserts without seeded cells were transferred to the wells. Coculture was maintained for 48 h. This duration was selected based on our migration and biodistribution analyses (Figure 2), which demonstrated that intravenously administered cells remain in the brain for at least 48 h. After 48 h, the Transwell inserts were removed, and the culture medium was replaced with DMEM/F12 containing 2% B-27 and 1% GlutaMAX™. The cells were maintained at 37°C in a humidified atmosphere with 5% CO₂, with medium changes every two days. Two weeks after the end of the co-culture period, the NSCs were fixed with 3% paraformaldehyde containing 1% sucrose, 1 mmol/L MgCl₂, and 0.1 mmol/L CaCl₂ for 30 min at room temperature. The fixed cells were incubated overnight at 4°C with primary antibodies diluted in PBS containing 4% donkey serum and 0.1% Triton X-100. The primary antibodies included mouse anti-MAP2 (1:100, Millipore) and rabbit anti-PSD95 (1:100, Abcam). After washing, the cells were incubated for 1 h at room temperature with Alexa Fluor-conjugated secondary antibodies: donkey anti-mouse IgG 488 and donkey anti-rabbit IgG 555. The nuclei were counterstained with Hoechst® 33342 (1:300, DOJINDO LABORATORIES, Kumamoto, Japan) for 30 min at room temperature.

Representative images were captured using a confocal laser scanning microscope (Ti-E A1R, Nikon Corporation, Tokyo, Japan). Confocal images were captured using a 60× water-immersion objective lens. No digital zoom was applied in any case. Image analysis was performed using Fiji software (ImageJ distribution, NIH, Bethesda, MD, USA).

REFERENCES

1. Koblan LW, Doman JL, Wilson C, Levy JM, Tay T, Newby GA, et al. Improving cytidine and adenine base editors by expression optimization and ancestral reconstruction. Nat Biotechnol. 2018;36:843-6.

2. Tanaka H, Miyama R, Sakurai Y, Tamagawa S, Nakai Y, Tange K, et al. Improvement of mRNA Delivery Efficiency to a T Cell Line by Modulating PEG-Lipid Content and Phospholipid Components of Lipid Nanoparticles. Pharmaceutics. 2021;13.

3. Kitase Y, Sato Y, Ueda K, Suzuki T, Mikrogeorgiou A, Sugiyama Y, et al. A Novel Treatment with Stem Cells from Human Exfoliated Deciduous Teeth for Hypoxic-Ischemic Encephalopathy in Neonatal Rats. Stem Cells Dev. 2020;29:63-74.
