# Supplementary material for: Novel stem cell therapy for cerebral palsy using stem cells from human exfoliated deciduous teeth
Source: Stem Cell Res Ther. 2026 Jan 23;17:44. doi: 10.1186/s13287-025-04828-y (PMC12833939; doi:10.1186/s13287-025-04828-y)
Supplement: Supplementary file 2 — Supplementary Material 2. [file 13287_2025_4828_MOESM2_ESM.docx]

**Additional File 2.**

**Supplemental Table1. Hindlimb Gait Scoring System in the Horizontal Ladder Test**

| **Score** | **Gait Description** |
| --- | --- |
| 0 | Plantar paw placement (normal stepping) |
| 1 | Slight slip during stride |
| 2 | Step with good paw replacement |
|  | Step with slight slip during paw replacement |
|  | Jumping step |
|  | Deep slip followed by successful paw replacement |
| 3 | Deep slip followed by a jump |
|  | Deep slip with unsuccessful or no paw replacement |
|  | Deep slip with a slight slip during paw replacement |
|  | Step with unsuccessful paw replacement |
| 4 | Paw placement absent |
|  | Fall |
|  | Collapse |
