# Supplementary material for: Novel stem cell therapy for cerebral palsy using stem cells from human exfoliated deciduous teeth
Source: Stem Cell Res Ther. 2026 Jan 23;17:44. doi: 10.1186/s13287-025-04828-y (PMC12833939; doi:10.1186/s13287-025-04828-y)
Supplement: Supplementary file 3 — Supplementary Material 3. [file 13287_2025_4828_MOESM3_ESM.docx]

**Additional File 3.**

**Supplementary Figure 1. Genome editing of HGF in SHED cells using ancBE4max**


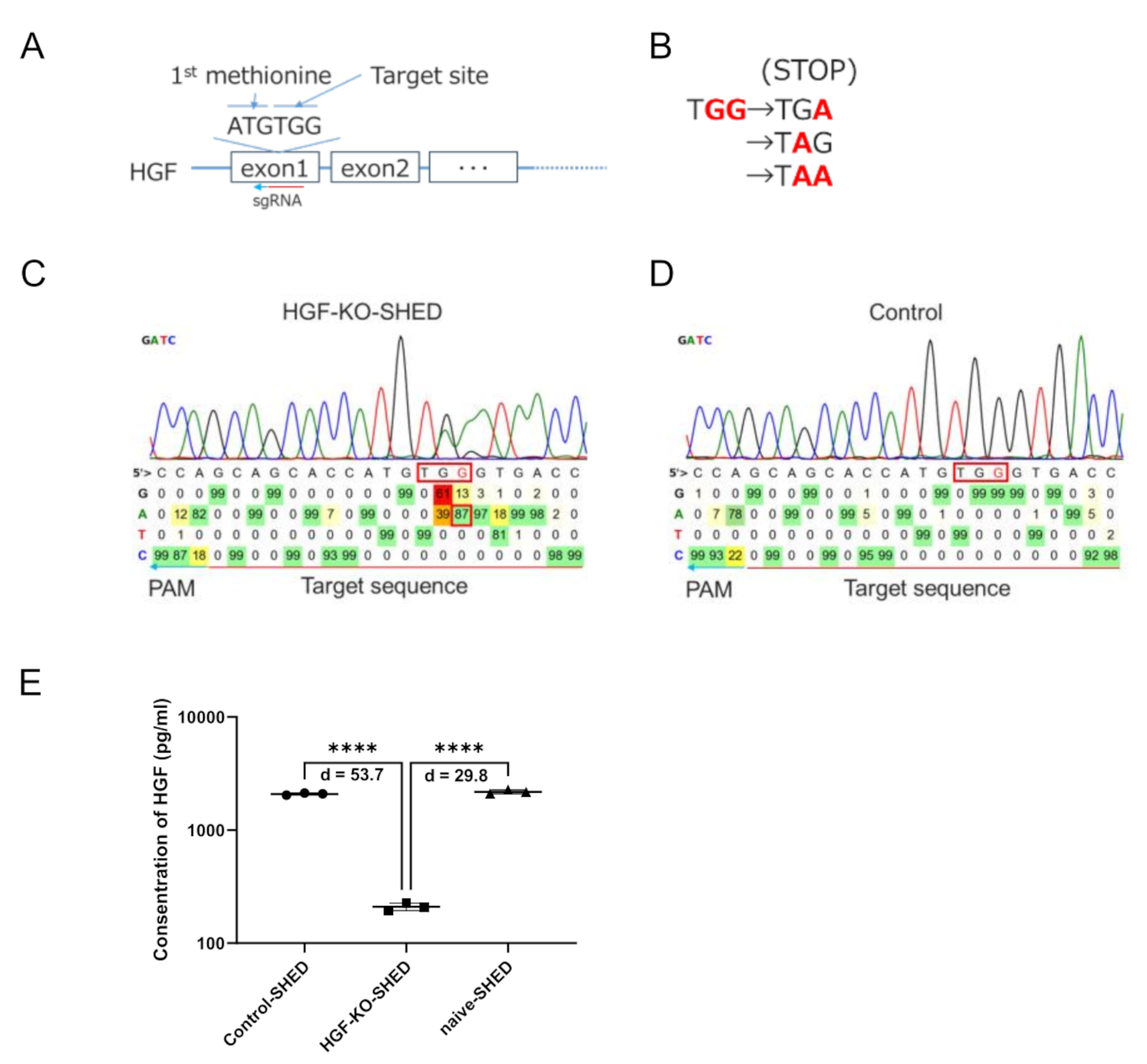

(A) A reverse-strand sgRNA was designed to target a codon adjacent to the first methionine in exon 1 of the *HGF* gene. The blue arrow and red line indicate the PAM sequence and the target region, respectively. (B) For the target codon “TGG,” guanine bases (GG) were editable by ancBE4max when directed by the reverse-strand sgRNA. Any permissible C-to-T conversions at these positions would result in a stop codon. (C, D) Evaluation of base editing efficiency by Sanger sequencing as analyzed with BEAT. (C) SHED cells transfected with ancBE4max mRNA and *HGF*-targeting sgRNA. (D) SHED cells transfected with ancBE4max mRNA and a non-targeting control sgRNA. The analysis revealed that at least 87% of the alleles in the targeted sample acquired a stop codon mutation, whereas no editing was observed with the nontargeting sgRNA. (E) The mean concentration of HGF in each conditioned medium was measured using the LEGENDplex™ Human Growth Factor Panel. The data are presented as the mean ± SD (n = 4). ****p <0.0001 by one-way ANOVA with Holm–Šídák’s multiple comparisons test. Values of Cohen’s d are indicated in the graph to represent the effect sizes of group differences.
