# Supplementary material for: Novel stem cell therapy for cerebral palsy using stem cells from human exfoliated deciduous teeth
Source: Stem Cell Res Ther. 2026 Jan 23;17:44. doi: 10.1186/s13287-025-04828-y (PMC12833939; doi:10.1186/s13287-025-04828-y)
Supplement: Supplementary file 4 — Supplementary Material 4. [file 13287_2025_4828_MOESM4_ESM.docx]

**Additional File 4.**

**Supplementary Figure 2. Assessment of Apoptosis Following SHED Administration in a Cerebral Palsy Model**


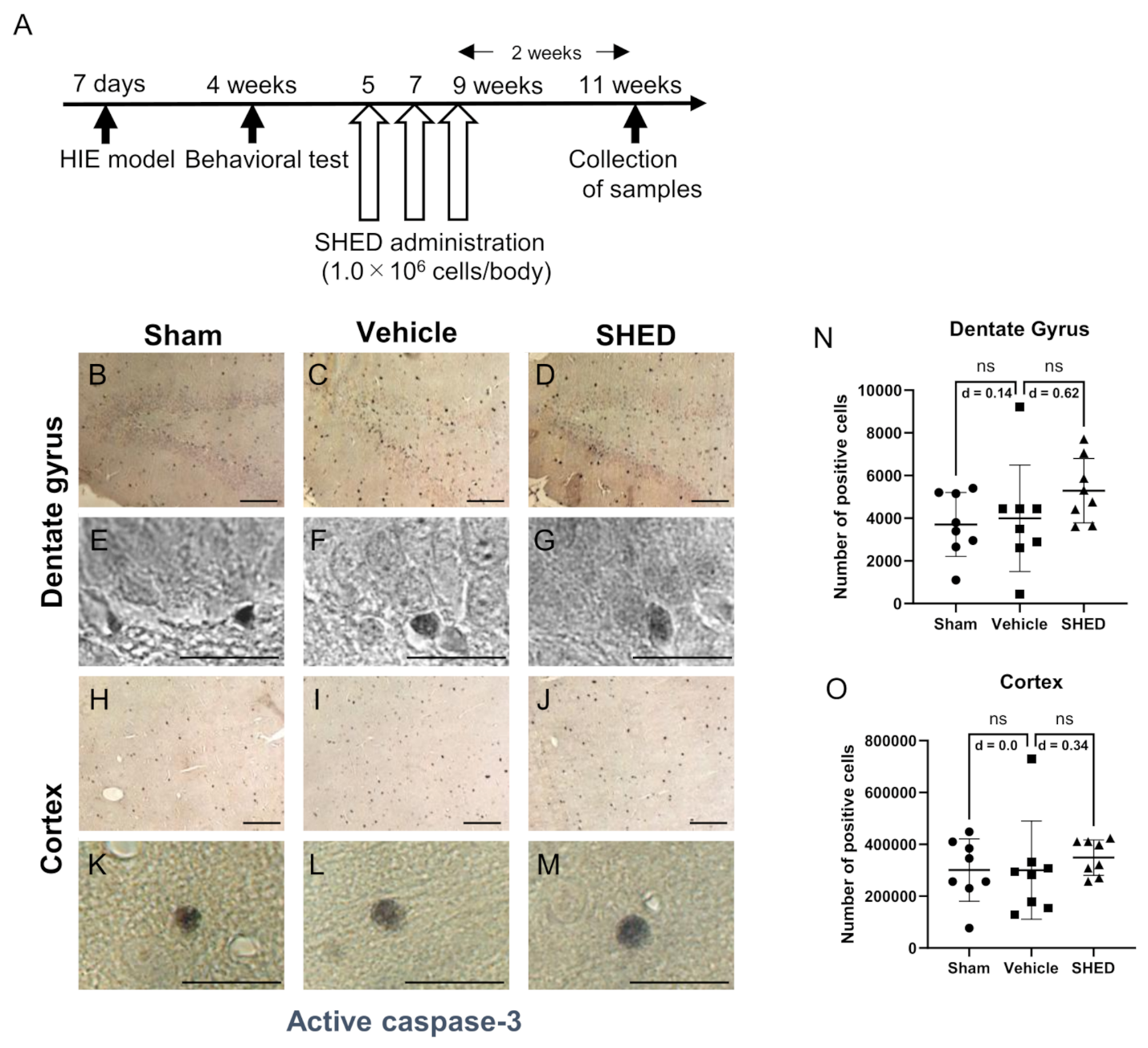


(A) Timeline of SHED administration and sample collection for histological evaluation. (B–D) Representative images of active caspase-3 (blue) immunostaining in the hippocampal dentate gyrus of each group. Bar = 100 μm (E–G) Representative images at a higher magnification for each group. Bar = 20 μm (H–J) Representative image of active caspase-3 immunostaining in the cortex of rats in each group. Bar = 100 μm (K–M) Representative image at a higher-magnification view for each group. Bar = 20 μm (N, O) Average Number of active caspase-3-positive cells in the hippocampal dentate gyrus or cortex of each group. The data are presented as the mean ± SD; Vehicle: n = 8, SHED: n = 8, Sham: n = 8); the one-way ANOVA with Holm–Šídák’s multiple comparisons test. Values of Cohen’s d are indicated in the graph to represent the effect sizes of group differences.
