# Supplementary material for: Novel stem cell therapy for cerebral palsy using stem cells from human exfoliated deciduous teeth
Source: Stem Cell Res Ther. 2026 Jan 23;17:44. doi: 10.1186/s13287-025-04828-y (PMC12833939; doi:10.1186/s13287-025-04828-y)
Supplement: Supplementary file 5 — Supplementary Material 5. [file 13287_2025_4828_MOESM5_ESM.docx]

**Additional File 5.**

**Supplementary Figure 3. Assessment of NSC Differentiation and Functional Network Integration after Coculture with SHED**


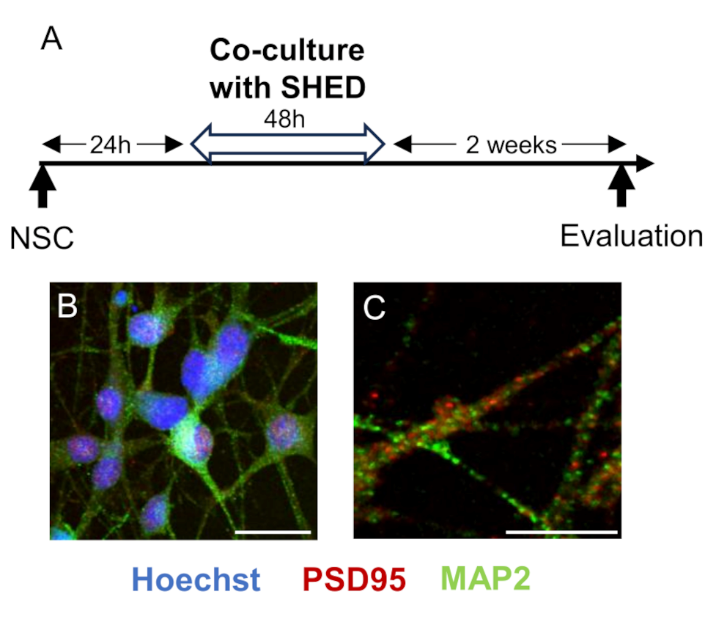

(A) Timeline of NSC differentiation experiments following coculture with SHED. (B) Representative images of MAP2 (green) immunostaining in NSCs following coculture with SHED. Scale bar = 25 μm. (C) Higher magnification images of MAP2 (green) and PSD95 (red) immunostaining in NSCs after coculture with SHED. Scale bar = 10 μm.
